# *De novo *assembly and characterization of the carrot transcriptome reveals novel genes, new markers, and genetic diversity

**DOI:** 10.1186/1471-2164-12-389

**Published:** 2011-08-02

**Authors:** Massimo Iorizzo, Douglas A Senalik, Dariusz Grzebelus, Megan Bowman, Pablo F Cavagnaro, Marta Matvienko, Hamid Ashrafi, Allen Van Deynze, Philipp W Simon

**Affiliations:** 1 Department of Horticulture, University of Wisconsin, 1575 Linden Drive, Madison, WI 53706. USA; 2 USDA-Agricultural Research Service, Vegetable Crops Research Unit, University of Wisconsin, 1575 Linden Drive, Madison, WI 53706, USA; 3 Department of Genetics, Plant Breeding and Seed Science, Agricultural University of Krakow, Al. 29 Listopada 54, 31-425 Krakow, Poland; 4 CONICET and INTA EEA La Consulta, CC8 La Consulta (5567), Mendoza, Argentina; 5 Seed Biotechnology Center, University of California, 1 Shields Ave, Davis, CA, USA; 6 Genome Center, University of California, 1 Shields Ave, Davis, CA, USA; 7 Current address: Life Technologies, 850 Lincoln Center Circle, Foster City, CA, USA

## Abstract

**Background:**

Among next generation sequence technologies, platforms such as Illumina and SOLiD produce short reads but with higher coverage and lower cost per sequenced nucleotide than 454 or Sanger. A challenge now is to develop efficient strategies to use short-read length platforms for *de novo *assembly and marker development. The scope of this study was to develop a *de novo *assembly of carrot ESTs from multiple genotypes using the Illumina platform, and to identify polymorphisms.

**Results:**

A *de novo *assembly of transcriptome sequence from four genetic backgrounds produced 58,751 contigs and singletons. Over 50% of these assembled sequences were annotated allowing detection of transposable elements and new carrot anthocyanin genes. Presence of multiple genetic backgrounds in our assembly allowed the identification of 114 computationally polymorphic SSRs, and 20,058 SNPs at a depth of coverage of 20× or more. Polymorphisms were predominantly between inbred lines except for the cultivated x wild RIL pool which had high intra-sample polymorphism. About 90% and 88% of tested SSR and SNP primers amplified a product, of which 70% and 46%, respectively, were of the expected size. Out of verified SSR and SNP markers 84% and 82% were polymorphic. About 25% of SNPs genotyped were polymorphic in two diverse mapping populations.

**Conclusions:**

This study confirmed the potential of short read platforms for *de novo *EST assembly and identification of genetic polymorphisms in carrot. In addition we produced the first large-scale transcriptome of carrot, a species lacking genomic resources.

## Background

The most widely cultivated member of the *Apiaceae*, carrot (*Daucus carota *var. *sativus *L.) is a diploid (2*n *= 2*x *= 18) with a relatively small genome of 480 Mb. It has a history of cultivation as a root crop that dates back about 1100 years to Afghanistan [1]. Today, carrot is the largest single source of provitamin A carotenoids in the U.S. diet [2], and it is among the top ten vegetable crops globally in term of area of production and market value [http://faostat.fao.org/faostat/, [1,3]]. World carrot production is increasing and this has, in part, been attributed to an increased awareness of health benefits associated to carrot consumption by consumers [4].

Phenotypic and molecular diversity of carrot is expansive [5] and this diversity has been important in improving nutritional value and consumer quality; disease and pest resistance; and yield characteristics important for growers. Carrot genetic linkage maps have primarily been developed with anonymous AFLPs and RAPDs that require no prior genomic information, but these techniques yield primarily dominant markers [6]. Sequence-tagged codominant markers have been developed to facilitate selection for several traits, including major genes for carotenoid accumulation [7], sugar type [8], root-knot nematode resistance [9], and for 22 genes in the carotenoid biosynthetic pathway targeted for candidate gene analysis [10]. Although the carrot plastome has been sequenced [11], a 17-fold bacterial artificial chromosome library (BAC) has been developed and characterized [12], and microsatellite markers are being developed (unpublished data, Cavagnaro et al.), only relatively modest genomic resources have been developed for carrot [13].

ESTs have been a valuable resource to develop SNP (Single Nucleotide Polymorphism) and SSR (Simple Sequence Repeat) markers for many plants and animals, but as of Feb 22, 2011, only 2,914 non-organellar DNA sequences of *Daucus carota *are available in GenBank. An expansion of genomic sequence resources for carrot will be useful for a broad range of carrot genomic applications including the development of co-dominant markers such as SNPs and SSRs for genome mapping and diversity assessment.

The development of ESTs has historically relied upon Sanger sequence with some recent efforts also using longer-read next-generation sequencing technology such as Roche 454. Next generation sequence (NGS) technologies are revolutionizing molecular biology by lowering the cost per sequenced nucleotide and increasing the throughput [14]. Short reads platforms such as Illumina and SOLiD produce higher coverage and lower cost per sequenced nucleotide, but due to their shorter reads the use of those platforms has usually been restricted to resequencing applications which rely on a reference sequence for assisting the assembly. In absence of a reference sequence, a computational *de novo *assembly approach is required. With increased read length from technologies such as Illumina, and the development of new computational tools, short reads can be assembled and used for transcriptome analysis [14]. Recently three *de novo *assemblies using sequence reads from Illumina technology have been developed and described for plants [15-17]. Currently there are few genomic sequence or ESTs available for carrot.

The objectives of this study were to sequence carrot ESTs using the Illumina platform and to characterize the carrot transcriptome to develop a molecular resource for marker development and gene identification. This represents an application of short read sequence technology for transcriptome assembly of a plant lacking extensive genomic molecular resources. This EST collection will provide a valuable resource for genetic, diversity, structural and functional genomic studies in carrot.

## Results

### Sequencing and assembly

To develop an overview of the carrot transcriptome and obtain an initial comparison of cultivated and wild carrot transcripts, normalized cDNA libraries were constructed from four sources: two orange unrelated inbred lines of European origin, B493 and B6274 with Imperator and Nantes root shapes, respectively, [18]; a purple/yellow inbred line B7262 derived from an intercross between purple Turkish and orange Danvers (European) carrots [19]; and a pool of F4 RILs derived from a cross between B493 and QAL, a wild carrot from North America. These pooled F4's are referred to as B493 × QAL and were derived from a single B493 × QAL F1 plant. Therefore at most two haplotypes are represented among these transcripts. A total of 18 044 high quality Sanger sequences were obtained from the B493 library to generate a total of 8,221,411 nt with an average length of 456 nt. The three other libraries, B6274, B7262 and B493 × QAL were sequenced with Illumina GAII platform with 61 cycles to yield from 34 M to 39 M usable reads of 41 nt or longer for each genotype.

**Figure 1:**
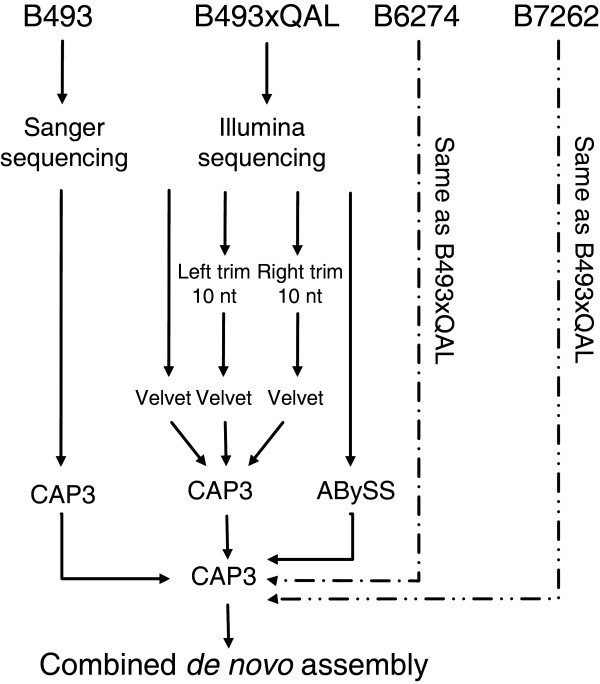
**Assembly strategy**. B493 Sanger sequences were assembled using CAP3. Two assemblies were generated for Illumina sequences for each genotype (B493xQAL, B6274 and B7262). After left and right Velvet trimming, sequences were assembled using CAP3. A second assembly was carried out using the ABySS short read assembler. A final CAP3 assembly allowed comparison to be made among sequences from all 4 genotypes to generate a combined *de novo *assembly.

**Figure 2:**
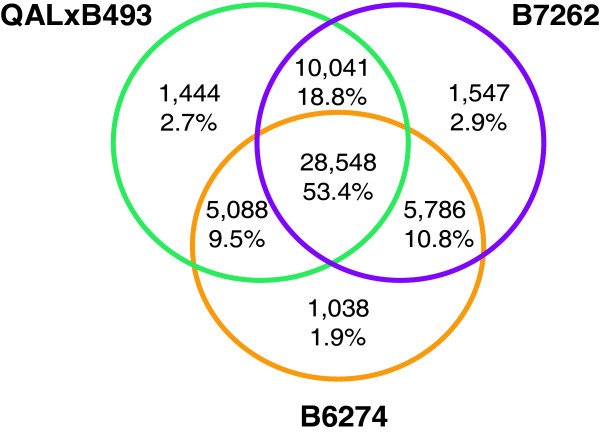
**Contig distribution among genotypes**.

A CAP3 [20] assembly of B493 Sanger sequences produced 4044 contigs plus 3241 singletons (Additional file 1, Table S1). A multiple step assembly strategy was used to produce a *de novo *assembly of the three Illumina sequence sets (Figure 1). For each genotype two separate assemblies were produced using either Velvet [21] combined with CAP3, or ABySS [22] (Additional file 1, Table S1). The Velvet+CAP3 assembly gave 31,337, 34,218, and 39,901 contigs for B6274, B7262, and B493 × QAL, respectively. The number of contigs produced by ABySS assembly was higher, ranging from 133,933 in B6274 to 193 844 for B493 × QAL. To combine the four sequences sources (B493, B493 × QAL, B6272 and B7262), a combined CAP3 assembly was created of contigs ≥100 nt. This cut off was selected based on annotation frequency vs. contig length (Additional file 1, Figure S1). The resulting sequence assembly produced 57,840 contigs plus 911 Sanger singletons with a total sequence length of about 45 Mb (Additional file 1, Table S2 and additional file 2). The average length of the contigs and singletons was 768.2 nt and the N50 (the contig lengths for which 50% of the sequence in an assembly is in contig of this size or larger) was 1378 nt. Out of the 58,751 contigs and singletons, 6,912 (11.7%) contained B493 Sanger sequences (Additional file 1, Figure S2). Among the Illumina-sequenced genotypes, B7262 sequences were most common in contigs, represented in 50,057 contigs (85.2%). Comparing Illumina-sequenced transcriptomes, a total of 19,762 contigs (38.1%) contained reads from only two genotypes, with 18.3% of the contigs having reads from B493 × QAL and B7262, 9.4% from B493 × QAL and B6274, and 10.4% from B7262 and B6274 (Figure 2). More than 50% of the assembled contigs contained sequences from all three genotypes. B7262 had the highest number of genotype specific contigs (1,494, 2.9%), and B6274 had the lowest with 1,017 (2.0%) genotype specific genes.

To test the quality of the assembly, we compared 20 full-length carrot mRNA sequences available from NCBI as references (Additional file 1, Figure S3). Corresponding *de novo *contigs were located using a BLASTN search, and a single best match for each *de novo *contig was found for each of the 20 reference genes. Raw Illumina and Sanger reads from each genotype were mapped onto each reference sequence and its corresponding *de novo *contig. All reference sequences were well covered by raw reads except at the ends, with 3' and 5' regions having relatively low coverage. Five of these 20 sequences were partially covered (353 to 675 nt) by B493 Sanger reads (data not shown). The average coverage among three Illumina sequenced-genotypes ranged from 32 (*DCMY*B) to 660 (*DcPRP*) reads. Fifteen of the 20 Sanger-derived reference cDNAs were 100% covered by Illumina reads. Of those, four were somewhat longer (40-100 base pairs) than reference sequences, perhaps due to differences in trimming of reads and coverage. The remaining five were partially covered by raw Illumina reads.

### EST annotation

**Figure 3:**
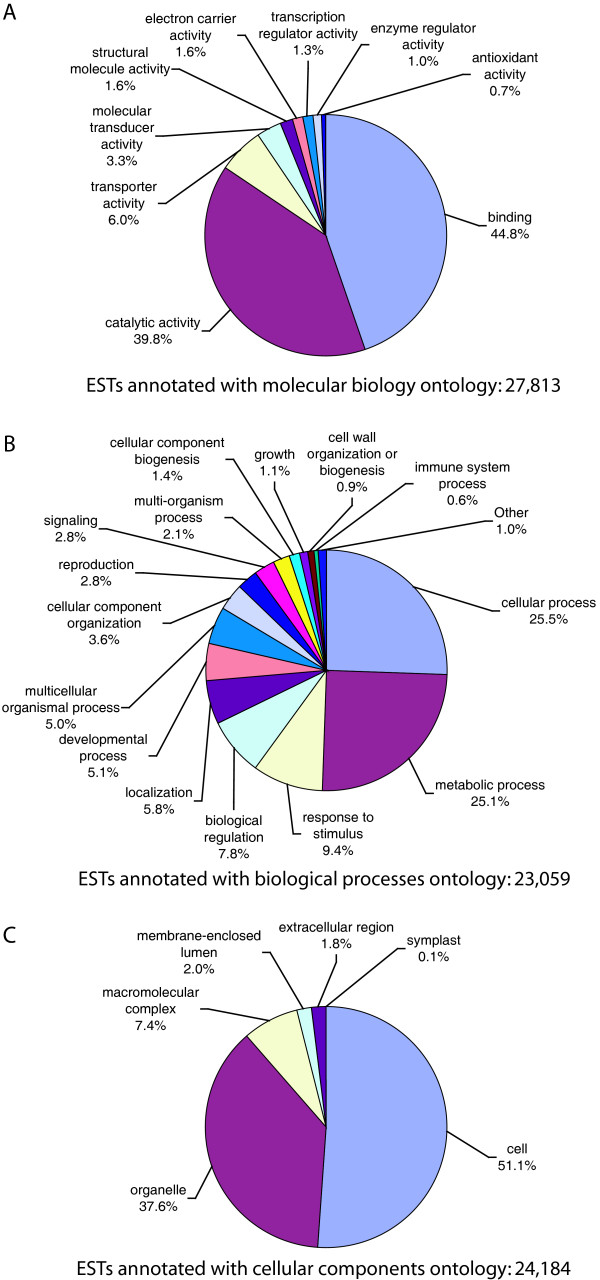
**Gene ontology distribution**. Gene Ontology distribution of the carrot ESTs derived from Blast2GO. The results are summarized as follows: (A) molecular functions; (B) biological processes; (C) cellular components

Consensus sequences were compared to the NCBI non-redundant nucleotide database using BLASTX with a cutoff e-value of e-05 and a length of coverage ≥33 aa. In total, 38,945 (67%) of the contigs had significant hits with known genes. Furthermore, we searched our EST collection using TBLASTN against all available *Daucus *protein sequences greater than 33 aa in GenBank to find contigs covering 80% of protein length. Out of 1196 of the proteins, 944 (78%) had a match with one or more of our contigs. BLAST2GO was used to assign gene ontology (GO) annotation. In total, 32,649 ESTs (55.6%) were annotated and classified into the three main GO categories: molecular functions, biological processes, and cellular components. As a result, 27,813 contigs were grouped under molecular functions, 23,059 under biological processes and 24,184 under cellular components. Among molecular functions, binding represented the most abundant category (44.8% of the contigs) followed by catalytic activity (39.8%) and transport activity (6.0%) (Figure 3A). Out of 23,059 contigs annotated as biological processes, 25.5% were annotated in the cellular process category, 25.1% in the metabolic process category, 9.4% in the response to stimulus category and 7.8% in biological regulation (Figure 3B). Contigs associated with cell (51.1%) and organelle (37.8%) categories represented the most dominant group of cellular component (Figure 3C).

As purple specialty carrots are a rich source of anthocyanins [23], and our EST collection included expressed genes from a purple carrot (B7262), we examined the expression of candidate genes in the anthocyanin pathway. Twelve gene families, represented by 21 published sequences, were compared to our assembly using BLASTN. This search revealed 9 contigs with significant matches to 5 gene families and to all the carrot anthocyanin reference genes from GenBank *(DcPAL1*, *DcPAL3*, *DcPAL4*, *CHS1*, *CHS3*, *CHS9, F3H*, *DFR1*, *DFR2*, *LDOX1 *and *LDOX2*), (Table 1). In addition, five contigs in three gene families represent genes previously not described in carrot. Two *phenylalanine ammoniam-lyase *genes (*PAL2 *and *PAL3*) described in *Petroselinum crispum *aligned with Contig21482 and Contig28113 (2,553 and 2,560 nt, respectively) with identities of 90 and 91%, respectively (Table 2). A *cinnamic acid 4-hydroxylase *(*CA4H*) gene from *Ammi majus *aligned with Contig524 (1798 nt) with an identity of 89%. In addition, two *4-coumarate coenzyme A ligase *genes (*4CL1 *and *4CL2*) from *P. crispum *aligned with Contig8294 and Contig52163 (1,814 and 1,937 nt long respectively). These results emphasize the depth of our EST database.

**Table 1:** Results of BLASTN of anthocyanin reference sequences against EST consensus sequences in this study

Gene family*	Reference sequence source	GenBank ID	Contigs with hits to reference
	*Daucus carota*	AB435640.1	Contig21709
*Phenylalanine ammoniam-lyase (PAL)*	*Daucus carota*	AB089813.1	Contig3962
	*Daucus carota*	D85850.1	Contig937
	*Petroselinum crispum*	X81159.1	Contig28113
	*Petroselinum crispum*	X81158.1	Contig21482
*cinammate 4-hydroxylase (CA4H)*	*Ammi majus*	AY219918.1	Contig524
*4-coumaroyl-coenzyme A ligase (4CL)*	*Petroselinum crispum*	X05350.1	Contig52163
	*Petroselinum crispum*	X05351.1	Contig8294
*Chalcone synthase (CHS)*	*Daucus carota*	Q9ZS40	Contig16173
	*Daucus carota*	Q9ZS41	Contig45955
	*Daucus carota*	Q9SB26	Contig16173
*Chalcone--flavonone isomerase (CHI)*	*Callistephus chinensis*	Q42663	-
*flavanone 3-hydroxylase (F3H)*	*Daucus carota*	AF184270.1	Contig52090
*Flavonoid 3'-monooxygenase (F3'H)*	*Petunia x hybrida*	Q9SBQ9	-
*flavonol synthase (FLS)*	*Petroselinum crispum*	AY230249.1	-
*Flavonoid 3',5'-hydroxylase (F3'5'H)*	*Campanula medium*	O04773	-
*Dihydroflavonol 4-reductase (DFR)*	*Daucus carota*	AF184272.1	Contig47011
	*Daucus carota*	AF184271.1	Contig3218
*leucoanthocyanidin dioxygenase 2 (LDOX)*	*Daucus carota*	AF184274.1	Contig22451
	*Daucus carota*	AF184273.1	Contig22451
*Anthocyanidin 3-O-glucosyltransferase (UFGT)*	*Gentiana triflora*	Q96493	-

* based on information from Hummer and Schreirer and Boss et al. [4,23]

**Table 2:** Previously unreported carrot anthocyanin genes

Gene	Reference sequence source	Carrot EST contig	Contig length (nt)	e-Value	BLASTN identities
PAL2	*P. crispum*	Contig21482	2,553	<1.00E-180	1,923/2,127 (90%)
PAL3	*P. crispum*	Contig28113	2,560	<1.00E-180	1,940/2,122 (91%)
CA4H	*A. majus*	Contig524	1,798	<1.00E-180	1,408/1,567 (89%)
4CL1	*P. crispum*	Contig8294	1,814	<1.00E-180	1,549/1,796 (87%)
4CL2	*P. crispum*	Contig52163	1,937	<1.00E-180	1,601/1,858 (87%)

Transposable elements (TEs) play an important role in shaping plant phenotypes, including an invertase mutant in carrot that conditions the type of sugar in storage roots [8,24]. In order to identify ESTs potentially originating from TEs, we selected 360 contigs annotated by BLAST2GO as TE-related. They were queried against RepBase ver. 15.12 (Dec 18, 2010) using Censor [25]. The resulting file (Additional file 3, Table S3) was manually searched for contigs with over 100 nt similarity to a particular superfamily and these contigs were subsequently assigned to that superfamily based on the classification system proposed by [26]. This resulted in the classification of 204 TE-related contigs. Transcripts related to Class I elements were four times more abundant than those of Class II (163 and 41 contigs, respectively). ESTs related to LTR retrotransposons, *gypsy*-derived transcripts were the most abundant. Transcripts originating from non-LTR retroelements and DNA transposons of the TIR order were also identified, while no *Helitron*-related transcripts were observed. ESTs attributed to *hAT*, *PIF/Harbinger*, and *CACTA *superfamilies were three times more abundant than those from *Tc1/Mariner *and *Mutator *superfamilies.

We also searched EST sequences for regions derived from known carrot Class II transposable elements, namely *DcMaster*-like, including *Krak *MITEs [27], a family of *Stowaway*-like MITEs - *DcSto *(unpublished data, Grzebelus et al.), non-autonomous *hAT *elements - *Dc-hAT1 *(unpublished data, Grzebelus et al.), and a family of *CACTA *elements - *Tdc *[28] (Additional file 1, Table S4). Only for the latter group of TEs could transposase-specific transcripts be detected. Three ESTs represented *Tdc*A1 transposase, while five others were likely chimeric transcripts containing portions of the transposase fused with other coding sequences (Additional file 3, Table S5). Only one of the *Tdc1 *transposase-specific ESTs (Contig3495) was identified as *CACTA*-derived in the general screen described above. In contrast, *DcMaster *transposase-specific transcripts were not detected, even though in the general screen 13 ESTs were attributed to the *PIF/Harbinger *superfamily. All hits found for *DcMaster/Krak*, *DcSto*, and *Dc-hAT1 *elements originated from non-coding regions of non-autonomous elements. *DcSto *elements were the most abundantly represented in the ESTs, including three transcripts containing complete copies of the MITEs. In a number of ESTs, regions derived from the non-autonomous TEs were present near the 5' or 3' end of the functional protein-coding transcript (Additional file 3, Table S6), suggesting that they were inserted in the 5' or 3'UTRs.

### Polymorphism detection

In total, 92% of the contigs contained sequences from at least two genotypes, which make the assembly a suitable resource for detecting candidate polymorphic markers such as SNPs, SSRs, and Indels (insertions or deletions).

We identified 8823 putative SSRs in 6,995 contigs (11.2% of the total 58,751 contigs) with 1,394 contigs containing two or more SSRs. A total of 323 SSRs were categorized as compound repeats. Repeat numbers ranged from 3 to 28 and length ranged from 12 to 84 nt. Trimers were the most abundant repeat motif accounting for 4,196 (47.6%) of the SSRs. Hexamers were the least common motifs with 527 (6%) of the SSRs (Additional file 1, Table S7).

Based on sequence alignments, we identified *in silico *polymorphic SSRs among the four genotypes. We detected 114 candidate polymorphic markers with SSR allele difference ranging from 3 nt to 14 nt. Trimers were confirmed to be the most abundant motif with 85 (75%) of the polymorphic SSRs (data not shown). Pentamers were the least common motif with 1 polymorphic SSR.

**Figure 4:**
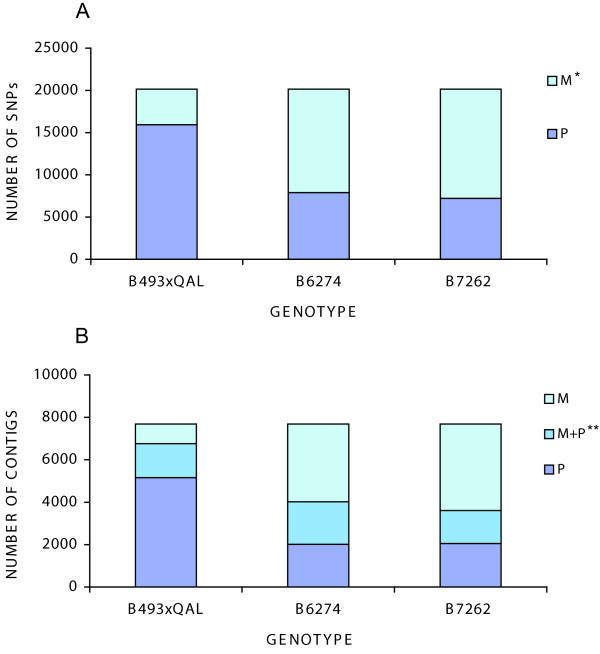
**Intra and inter-sample SNP distribution**. Intra- and inter-sample polymorphism distributions of computationally detected SNPs within carrot genotypes (B493xQAL, B6274 and B7262) at a depth of sequence coverage of 20 or more. The two graph reports: (A) distribution of intra and inter-sample polymorphism SNPs within genotypes; (B) distribution of intra and inter-sample polymorphism SNP within genotypes and contig containing SNPs. * M = intra-sample monomorphic, inter-sample polymorphic; P = intra- and inter-sample polymorphic. ** M+P = contigs containing both P and M SNP categories.

Mosaik software was used to detect SNPs. Due to the lower coverage of B493 Sanger sequences, detection of SNPs was performed using only into B493 × QAL, B6274 and B7262 datasets. At the level of stringency described in Materials and Methods, computational analysis allowed the detection of 20,058 SNPs in 7,684 contigs (13.2% of the total contigs) with 4,578 contigs containing two or more SNPs and an average number of 2.6 SNPs per contig and 1.36 SNPs per kb. Of the 20,058 SNPs, 13,756 (69%) were transitions (A/G, C/T) and 6,302 (31%) were transversions (G/T, C/G, A/T, A/C). Allele variation within each genotype was categorized by "M" for intra-sample monomorphic, inter-sample polymorphic SNPs, or "P" for SNPs that were polymorphic in both intra- and inter-sample comparisons. Intra-genotype polymorphism was highest for cultivated × wild carrot (B493 × QAL) RILs, and inter-genotype SNP polymorphism was greatest in comparison of B493 × QAL to the 2 cultivated carrot inbreds evaluated (Figure 4A). Expressing SNP categories relative to the total number of contigs, 11.5% (6,759) of B493xQAL contigs contain intra and inter polymorphic SNPs, B6274 contains 6.8% (4020), and B7262 6.1% (3609) (Figure 4B). This confirms the lower level of heterozygosity in inbred lines compared with a pool of the cultivated x wild carrot RILs. Considering the distribution of intra and inter-sample polymorphisms, the most abundant class of SNPs (35%) were those contrasting B493 × QAL and the two cultivated genotypes (PMM) (Additional file 1, Figure S4). Those represent an important SNP resource to further evaluate polymorphism between wild and cultivated germplasm.

### Marker validation

Marker validation was carried out on genomic DNA of the B493 × QAL wild carrot derivatives and three cultivated genotypes, B493, B6274 and B7262. Primers were designed and tested for 114 SSRs (1.3% of the total) identified computationally as polymorphic SSRs. Of these, 102 primers (89.5%) produced an amplification product (Table 3), with 99 of these producing a single product, and the remaining 3 SSRs have multiple products based on agarose gel resolution. Out of those 99 single products, 75 were of the expected size, and 24 were larger than the expected size. To verify the expected polymorphisms, 31 SSR products of expected size generated from the genotypes used in this study were resolved in a capillary system using fluorescent detection, and 26 of them were polymorphic while 5 were monomorphic.

**Table 3:** Evaluation of SSR primer pairs in four carrot genetic stocks used to develop the EST library

PCR results	Number of primers	Percentage of all primer tested	Percentage of amplified primer
**SSR**			
Tested	114		
Amplified	102	89.5	
Single product	99	86.8	97
Single expected product	75	65.8	74
Single larger product	24	21.1	24
Multiple product	3	2.6	3
Polymorphic*	26		

*out of 31 tested.

To validate SNPs, 354 primer pairs were designed and tested, of which 311 SNPs (88%) produced an amplification product (Table 4). Of these, 272 primer pairs produced a single product, and 39 gave multiple products. Out of those 272 single products, 162 were of the expected size, and 110 were larger than expected size. SNPs were verified by sequencing all 162 single products with expected size, and 96 of those single products of larger size, but shorter than 500 nt. Among these 258 putative SNP sequences, 212 had the expected SNPs. Thus, 60% of the SNPs tested and 82% of those that amplified a single product shorter than 500 nt were polymorphic and useful for mapping.

**Table 4:** Evaluation of SNP primer pairs in four carrot genetic stocks used to develop the EST library

PCR results	Number of primers	Percentage of all primer tested	Percentage of amplified primer	Percentage of amplified and sequenced primers
**SNP**				
Tested	354	-	-	-
Amplified	311	88	-	-
Single product	272	77	87	-
Single expected product	162	46	52	-
Single larger product	110	31	35	-
Multiple product	39	11	13	-
Sequenced*	258	73	83	-
Polymorphic	212	60	68	82

*Considering 162 single expected product and 96 single larger product whit amplicon size <500 nt

### Intron predictions

Primer pairs that amplify a larger than expected products are presumed to be the result of an intron between the two primer sites. To evaluate how we could reduce the number of markers in this category which complicate SNP primer design, we predicted introns for the 20,148 SNPs using the Solanaceae Genomics Network Intron Finder, which uses an *Arabidopsis *database (Additional file 1, Table S8). The number of SNPs with no predicted nearby intron was 11,183 (55.5%), in 4,814 contigs. We compared our SNP PCR validation rate with this intron prediction. Of the 354 designed and tested primers, 46% (162) gave a single expected product (SEP) (see SNP validation above and Table 4). Computational intron prediction found 120 SEP primer pairs, which would be candidate for primer design (Additional file 1, Table S8). Of this 91 (76%) were validated by PCR. That prediction would also have rejected 71 [30% (162 minus 91)] useful SEP category. Nevertheless, in our case, the use of this intron-prediction tool would have increased success rate of single expected products from 46% to 76%. Using this approach, primer design and contig detection can be improved to optimize SNP discovery.

### SNPs polymorphism within mapping populations

The 212 polymorphic SNP markers were screened in two mapping populations; B493 × QAL [29], which had alleles identified from this EST project, and an unrelated mapping population 70349 (unpublished data, Cavagnaro et al.). Ten genotypes from each mapping population were screened by PCR and all amplicons sequenced, thus allowing identification of polymorphic markers. In total, 48 polymorphic markers (23%) were identified in the B493 × QAL population and 50 (24%) were polymorphic in the 70349 mapping population (Additional file 1, Table S9), with 11 polymorphic markers common to both populations. Thus, a total of 87 polymorphic markers were identified. Overall, 41% (87/212) of the SNP markers that were polymorphic among the genotypes used for developing the EST datasets, and 28% (87/311) of the primer pairs that produced amplicons were polymorphic in the two mapping populations. These markers have potential for mapping and as anchor points for carrot map merging.

## Discussion

Next generation sequencing technologies are revolutionizing molecular biology by lowering the cost per sequenced nucleotide, increasing the throughput and eliminating laborious bacterial cloning. Despite of the shorter sequence reads, compared to Sanger sequencing, the lower cost and high coverage of NGS are the two main factors that drive researchers to use these new technologies. Due to the longer reads produced by the Roche GS FLX technology, this NGS platform is most commonly used for *de novo *transcriptome sequencing. This platform has been used for transcriptome sequencing of pine [30], oats [31], *Aegilops *[32] and buckwheat [33]. In contrast, short read-length platforms such as Illumina and SOLiD, which produce higher coverage and lower cost per sequenced nucleotide, have been relegated to resequencing applications which usually depend, for their assembly, on a reference sequence. With the improvement of read length for technologies such as Illumina, and the development of new computational tools, we have demonstrated that short reads can be assembled and used for transcriptome analysis. Indeed, other recently *de novo *transcriptome assemblies using Illumina sequences have been successfully developed and described in *Ipomoea *[15], whitefly [34], *Eucalyptus *[16] chickpea [17] and orchids [35]. Consistent with previous work, our results demonstrate that short reads can be assembled and used for transcriptome analysis, gene identification and marker development in carrot. We assembled 58,751 contigs and singletons from 114 M Illumina reads and 18,044 Sanger sequences from four different genotypes.

Quality of the *de novo *assembly was confirmed by sequence comparison, annotation and marker validation. Comparison of assembled contigs with full-length cloned carrot gene sequences confirmed the high quality of the assembly. Seventy-five percent of the contigs aligned nearly completely with mRNA reference sequences. These results were similar to those previously obtained by Mizrachi and colleagues [16] in *Eucalyptus*. Distributions of genotype-specific contigs in the different EST datasets of carrot were similar, with B493 × QAL and B7262 showing the highest number of reads in common. In addition, only 2.3% of all the EST contigs were unique to the wild x cultivated (B493 × QAL) dataset. Altogether, these results suggest that the wild carrot transcriptome is not significantly different from the cultivated carrot transcriptome, which is consistent with cross-ability among wild and cultivated carrot in *D. carota*.

About 67% and 55% of the contigs exhibited homology using BLASTX and BLAST2GO, respectively, indicating that contiguity of the sequence is consistent for most of the assembled transcriptome. BLAST2GO annotation indicated that a wide range of transcriptome diversity was included in the ESTs we evaluated. Contigs without significant matches to the existing databases could reflect either novel, carrot-specific genes or could reflect a poor representation of Apiales sequences in GenBank.

Manual annotation confirmed expression of 11 known carrot anthocyanin genes and allowed identification of five new ones. In addition to the three known carrot *phenylalanine ammonia-lyase *(*PAL*) genes, identification of two new *PAL *genes suggests that multiple -and diverse- members comprise this gene family in carrot. The five previously-unreported anthocyanin biosynthesis genes discovered in this study confirms the usefulness of this new molecular resource for discovering genes of carrot.

Transcripts related to transposable elements constituted 0.34% of the assembled contigs. As TE-related transcripts were initially identified on the basis of the BLAST2GO annotation, that estimate should be viewed as conservative. For example, no *Helitron*-related contigs were found in the carrot transcriptome, which might be due to the fact that few transcripts originating from *Helitrons *have been annotated in the GenBank database. Our observation of a range of functional TE transcripts suggests that members of many TE families could potentially be active in carrot. Earlier reports indicated that *Tdc *elements were activated in the course of long term *in vitro *cultures [36] and this along with our observations that *DcMaster *and related MITEs were highly polymorphic, likely suggests their very recent activity [24-27].

Several contigs contained MITE-derived regions, usually located close to the 5' or 3' ends. This was observed for both *Tourist*-like (*Krak*, *KrakL1*), *Stowaway*-like (*DcSto*), and *hAT*-like (*Dc-hAT1*) elements, indicating that MITEs in the carrot genome were associated with non-coding regions of genes, similar to their reported occurrence in grasses [37-39]. In contrast, *CACTA *elements did not show this pattern of insertion preference.

Verification of assembled contigs through PCR amplification represents a good test of quality of the assembly. The goals of this study were to use a multi-genotype based assembly to develop co-dominant molecular markers, such as Simple Sequence Repeats (SSRs) and Single Nucleotide Polymorphisms (SNPs). SSR trimers were the most abundant repeat motif, confirming previous observations by Cavagnaro and colleagues (unpublished data).

Use of multiple genetic backgrounds in our EST analysis allowed us to identify 114 computational polymorphic SSRs and 20,058 SNPs at a depth of coverage of at least 20. Most of the polymorphic SNPs found in carrot inbred lines were polymorphic when compared against other lines but were monomorphic within lines. This observation indicates that transcriptome comparison is an efficient strategy to identify SNP markers for molecular genetic mapping and diversity analysis. Within-sample SNP polymorphism in the cultivated × wild carrot (B493 × QAL) derivatives was highest (Figure 4). As QAL is expected to be heterozygous this sample was designed as a pool of RILs to represent alleles segregating in the B493 x QAL mapping population. SNP polymorphism (35%) between B493 × QAL and the two cultivated genotypes and among cultivated genotypes used in this study was also high. Although the gene content in wild and cultivated carrot was highly similar, there is a high degree of allelic variation among them.

Primers were designed and tested for 114 computational polymorphic SSRs and 354 SNP loci. Of these, we were able to amplify 102 SSRs (~90%) and 311 SNPs (~88%), with 27 and 110 markers, respectively, amplifying a product larger than expected, suggesting the presence of intron(s) within the amplicon. Validation rate (expected size rate and polymorphic rate) showed that our results were similar or higher than what was previously obtained in *Cajanus *[40], iris [41], *Epimedium *[42], *Pinus *[30], chickpea [17], *Cryptomeria *[43], apple [44], bean [45] and oat [31] where Sanger, 454, and Illumina platforms were used for sequencing.

To evaluate how intron prediction could affect SNP validation rate we predicted introns using the Sol Genomics Network Intron Finder *Arabidopsis *database. Based on our SNP validation data, intron prediction would increase the yield of single expected size PCR products from 46% to 76%. In contrast, due to the genetic distance between carrot and Arabidopsis, carrot specific regions would be excluded and decrease the total number of useful SNPs by about 20%. Our data suggests that for species unrelated to *Arabidopsis *it would be better to use both introns predicted and empirical data for assay design to maximize validation rate and evaluate genetic diversity.

In our evaluation of two mapping populations, the B493 × QAL population had alleles identified directly from the ESTs, whereas the second mapping population, 70349 was unrelated to our EST sequence data. Interestingly, about a 25% of the 212 SNPs evaluated were polymorphic in both mapping populations. About 13% of the SNPs were polymorphic in both mapping populations, the remainder being polymorphic in one population but not the other. This small-scale assay provides important information useful in predicting the number of markers to screen in designing high throughput molecular assays.

## Conclusions

In this study we confirmed the potential of using a short read sequencing platform for *de novo *assembly producing the first large-scale transcriptome sequence set of carrot a species lacking genomic resources. EST characterization provided evidence of the usefulness of this resource for gene detection and mapping of carrot. In addition we demonstrated that transcriptome comparisons provide an efficient strategy for marker development allowing detection and validation of computational polymorphic SSRs and a large set of SNPs.

## Methods

### Plant Materials

Carrot material for inbred lines, B6274, B7262, and B493, as well as the pool of F_4 _B493xQAL RILs were grown in pots under greenhouse conditions. Root and leaf tissues were harvested after ten weeks post planting, with the leaf tissue separated from the root immediately upon harvest. Both the leaf and the storage root tissues were flash frozen in liquid nitrogen and stored at -80°C.

### RNA Extraction

A CTAB based RNA extraction protocol modified from Chang and colleagues [46] was used to extract RNAs for both the Sanger and Illumna sequencing projects. For the pooled extraction of the F_4 _B493xQAL RILs, RNAs were extracted from the leaf tissue and root tissue of individual plants representing a variety of pigmented carrot root tissue and then pooled after extraction using equal amounts of mRNA from each tissue type and genotype. The extracted RNAs were analyzed for potential degradation by gel electrophoresis. RNA concentration was quantified using a NanoDrop spectrophotometer (NanoDrop Technologies). cDNA was synthesized and prepared for paired-end sequencing as described by Illumina [47]. Samples were sheared, 300-350 bp fragments selected, and were normalized using double-stranded nuclease that digests high copy double stranded DNA during re-association after denaturation. For Sanger sequencing, normalized cDNA libraries were constructed from root and leaf as described above for B493 with insert sizes ranging from 1.0-2.0 kb and above 2 kb and cloned into pDNR-LIB using the SMART(TM) cDNA library kit (Clontech, Palo Alto, CA).

### Sequencing and assembly

Illumina sequencing was performed with the GAII platform at the UC Davis Genome Center (University of California, Davis, USA) according to the manufacturer's instructions (Illumina, San Diego, CA). Twenty thousand reads were attempted with T7 primer on Sanger 3730 (Symbio Inc, Menlo Park, CA).

Details of assembly procedure are reported in additional file 4. Briefly, Sanger read basecalls and quality scores were made with phred version 0.020425.c [48]. Vector sequence and low quality bases were trimmed with Lucy version 1.19p [49]. Resulting sequences and quality files were assembled with CAP3 version 12/21/07 [20] with default parameters.

Three versions of the original Illumina reads from each genotype were created to be used for assembly: unmodified original reads (A); 10 base pairs trimmed (in order to remove low quality reads) sequences from the left (5') (B) and right (5') (C) Optimal kmer length was determined by assembling one genotype at all possible kmer values from 20 to 60 using Velvet version 0.7.55 [21]. The kmer length of 41 was selected for further assemblies. Each of the three Illumina read sets for each of the three genotypes was assembled separately. The resulting three assemblies within each genotype were merged by assembling with CAP3. Unmodified original reads (set A) were also assembled using ABySS version 1.0.15 [22]. Optimal kmer length was determined by performing assemblies on one genotype at a time and each of the three Illumina read sets was assembled separately. Resulting contigs and singlets from the CAP3 Sanger sequence assembly, Velvet+CAP3 Illumina sequence assemblies and ABySS Illumina sequence assemblies were assembled into a consensus assembly with CAP3, generating the reference *de novo *multi-genotype assembly. Illumina sequences generated in this study have been deposited in Data Bank of Japan (DDBJ) Sequence Read Archive (DRA, http://trace.ddbj.nig.ac.jp/dra/) with accession number SRA 035376. Sanger sequences have been deposited in NCBI Expressed Sequence Tags database (dbEST, http://www.ncbi.nlm.nih.gov/dbEST/) with accession number JG753039-JG771082.

### Contig validation

To verify quality of the assembly, 20 full-length carrot genes available in GenBank were used to map raw Illumina reads and align the correspondent *de novo *contigs. Alignment of reads against full-length reference sequences and correspondent contig was carried out using BLAST ver. 2.2.24+ (ftp://ftp.ncbi.nlm.nih.gov/blast/executables/blast+/LATEST/) with the following parameters: e-value: 10; dust filter; off; minimum blast hit length: 51 nt; minimum blast hit percent match: 80. A global pairwise alignment of the full-length reference sequence and corresponding contig was performed using the Needle program ver. 6.3.1 from the EMBOSS package [50].

### Homology search and functional annotation

Assembled sequences were used for blast searches and annotation against the NCBI nr database using a cutoff e-value of e-05 and minimum coverage length ≥33aa. *Daucus *protein sequences greater than 33 amino acids available in GenBank were used for blast (TBLASTX) analysis against our EST collection, using a cutoff value of e-05 and minimum coverage length ≥100 nt. Functional annotation and gene ontology term assignment was carried out using BLAST2GO (http://www.geneontology.org). In order to find ESTs potentially originating from anthocyanin genes, 21 complete sequences from GenBank were selected and blasted against our local database with a cutoff e-value of e-05. We also searched for ESTs potentially originating from transposable elements (TEs). We filtered contigs annotated as TE-related from the BLAST2GO output. They were queried against RepBase ver. 15.12 (Dec 18, 2010) using Censor [25].

In order to identify transcripts containing fragments of previously described carrot Class II transposons *DcMaster*, *Krak*, and *Tdc1*, together with unpublished MITEs *DcSto *and *Dc-hAT1*, their consensus sequences were used as blast queries against the EST database with e-value cutoff equal e-02.

### Identification of EST-SSRs and SNPs

SSR motifs were identified using MISA 1.0 (MIcroSAtellite; http://pgrc.ipk-gatersleben.de/misa/) [51], which identifies both perfect and compound repeats. We searched for di-, tri-, tetra-, penta- and hexa-nucleotide repeats with a minimum of six repeat units for dinucleotides, four for trinucletides and three repeat units for tetra, penta and hexanucleotides. Adjacent microsatellites ≤10 nt apart were considered compound repeats.

Polymorphic SSRs were detected computationally by a custom Perl program that analyzed the output of the final CAP3 assembly stage. Indels from 3 nt to 50 nt in size, and with at least 25 nt of flanking sequence were considered.

SNP detection was performed using Mosaik 1.0.1388 [52] with the following parameters: maximum hash positions per seed: 100; alignment candidate threshold: 20. This resulted in the detection of 346,456 SNPs. For marker validation and data analysis we reduced this to 20,148 SNPs using the following parameter: most abundant allele minimum read coverage: 10; second most abundant allele minimum read coverage: 10; minimum average coverage: 10; minimum flanking length: 100 nt; minimum quality score: 0.99; minimum absolute isolation: 30 nt.

### Primer design and amplification

Primer pairs flanking the SSR motifs were designed using MISA with the following parameters: end stability: 250; minimum size: 100 nt; maximum size: 300 nt. In total we synthesized 114 primer pairs (Additional file 3, Table S10).

Primer pairs flanking the SNPs were designed using Primer3 [53] with the following parameters: end stability: 250; optimum Tm: 60°C; minimum size: 120 nt; maximum size: 201 nt. In total we synthesized 354 primer pairs (Additional file 3, Table S11).

Primer validation was carried out on genomic DNA of the B493 × QAL wild carrot derivatives and three cultivated genotypes, B493, B6272 and B7262. Microsatellite flanking primers were tested in a PCR of 20 μl volume containing 13 μl water, 2 μl 10X DNA polymerase buffer, 0.8 μl dNTPs (2.5 mM each), 1 μl 5 μM of each primer, 0.2 μl *Taq *polymerase (MBI, Fermentas, USA) and 2 μl of genomic DNA (~50 ng). PCR conditions were: initial denaturation at 94°C for 2 min., followed by 33 cycles of 94°C for 45 sec., Tm (°C) for 1.0 min., 72°C for 1.0 min. and 20 sec., and a final step at 72°C for 10.0 min.. Electrophoresis was carried out for 2-3 hours at 100 V on 2% agarose TAE gels supplemented with 0.2 μg/ml of ethidium bromide.

To verify the predicted polymorphism of SSRs, 31 primer pairs were tested using a fluorescent method [54]. PCR was performed in a 20 μl final volume including 12.5 μl water, 2 μl 10X DNA polymerase buffer, 0.8 μl dNTPs (2.5 mM each), 1 μl 5 μM of reverse primer, 0.5 μl 5 μM of M13-tailed forward primer, 1 μl 5 μM of M13 labelled either with 6-FAM, HEX or NED fluorochromes, 0.2 μl *Taq *polymerase (MBI, Fermentas, USA) and 2 μl of genomic DNA (~50 ng). The amplification conditions were 94°C for 2 min; 10 cycles of 94°C for 40 sec, 60°C for 1 min with a reduction of 0.5°C each cycle, 72°C for 1 min; 40 cycle of 94°C for 45 sec., 60°C for 1.0 min., 72°C for 1.0 min. and 20 sec, and a final step at 72°C for 10.0 min. Amplicon lengths were estimated using an ABI 3730*xl *capillary sequencer available at the University of Wisconsin Biotechnology Center and analyzed with GeneMarker software version 1.5 (SoftGenetics, State College, Pennsylvania).

Single Nucleotide Polymorphism validation was carried out on a PCR of 20 μl volume containing 12.2 μl water, 2 μl 10X DNA polymerase buffer, 1.6 μl dNTPs (2.5 mM each), 1 μl 5 μM of each primer, 0.2 μl *Taq *polymerase (MBI, Fermentas, USA) and 2 μl of genomic DNA (~50 ng). PCR conditions were: initial denaturation at 94°C for 2 min., followed by 25 cycles of 94°C for 30 sec., appropriate annealing temperature (Table S4) for 30 sec., and 72°C for 45 sec., and a final step at 72°C for 10 min. Presence and length of the amplicon was detected on 2% agarose TAE gels supplemented with 0.2 μg/ml of ethidium bromide, and separated for 2-3 hours at 100 V. To verify presence of the expected SNP, single products of expected size and single products larger than expected, with an overall size shorter than 500 bp, were sequenced. Sequencing reactions were performed in a 5 μl final volume including, 1.75 μl of water, 1 μl of 5 μM primer, 0.75 μl 5 × BigDye^®^3.1 sequencing buffer, 0.5 μl of BigDye^®^3.1 ready reaction mix and 1 μl of PCR product, previously diluted 1:10 with water. Amplification conditions were: 25 cycles of 96°C for 10 sec., and 58°C for 2 minutes, and a final step at 72°C for 5.0 min. The sequences were generated by the University of Wisconsin Biotechnology Center and analyzed using Sequencher software version 4.8 (GeneCodes Corporation, Ann Arbor, MI).

### Intron prediction

Intron prediction was carried out using Intron Finder (http://solgenomics.net/tools/intron_detection/find_introns.pl) with a cutoff e-value of e-50. Intron prediction results for the 354 assembled contigs screened for SNPs detection, were compared with our validation data results.

### SNP polymorphisms within mapping populations

The *in silico *predicted polymorphic SNP markers were screened in two mapping populations including B493 × QAL [29] and 70349. Ten genotypes from each mapping population were screened on a PCR of 15 μl volume containing 12.2 μl water, 2 μl 10X DNA polymerase buffer, 1.6 μl dNTPs (2.5 mM each), 1 μl 5 μM of each primer, 0.2 μl *Taq *polymerase (MBI, Fermentas, USA) and 2 μl of genomic DNA (~50 ng). PCR conditions were: initial denaturation at 94°C for 2 min., followed by 25 cycles of 94°C for 30 sec., appropriate annealing temperature (Table S5) for 30 sec., and 72°C for 45 sec., and a final step at 72°C for 10 min. Quality of the amplicon was detected on 2% agarose TAE gels supplemented with 0.2 μg/ml of ethidium bromide, and separated for 2-3 hours at 100 V. To detect SNP polymorphism, PCR products were analyzed by sequencing as previously described.

## Abbreviations

AFLP: amplified fragment length polymorphism; EST: expressed sequences tag; PCR: polymerase chain reaction; SNP: single nucleotide polymorphism; QTL: quantitative trait loci; BAC: bacterial artificial chromosome; RAPD: random amplified polymorphic DNA; SSR: simple sequence repeat; NGS: next generation sequence

## Authors' contributions

MI detected anthocyanin gene homologs, tested and validated SNP and SSR primers, evaluated SNPs in two mapping populations, interpreted the data and wrote most sections of the manuscript; DAS carried out most of the bioinformatic analysis and revised the paper; DG: identified transposable element homologs within the ESTs and wrote the transposable element section; MB: prepared the RNA and wrote the relevant methods section; participated in data interpretation and revision of manuscript; PFC: participated in the EST-SSR analysis and related writing; MM: constructed the normalized cDNA libraries for Sanger and Illumina sequencing; wrote relevant methods sections, participated in data interpretation and revision of manuscript; HA: was involved in data interpretation and guidance for analysis; AVD: participated in the experimental design and initiation of the experiments, data interpretation, and revision of the manuscript; PWS: developed the plant material, participated in the experimental design and initiation of the experiments, data interpretation, writing and revision of several sections of the manuscript. All authors read and approved the final manuscript.

## Supplementary Material

Additional file 1 **Table S1 – Individual genotype transcriptome assemblies.** Summary of the number of contigs and singletons obtained for the B493, B493×QAL, B6274 and B7262 individual transcriptome assemblies using different assembly methods.     **Table S2 – Combined transcriptome assemblies.** Summary of the B493, B493×QAL, B6274, and B7262 combined transcriptome assemblies.    **Table S4 Transposable element superfamilies and families represented in ESTs.Table S7 - Distribution of motif length in the SSR dataset.Table S8 - Comparison of SNP validation rates using intron prediction.Table S9 - Polymorphic SNPs tested in two mapping populations.** Summary of results obtained by screening of two mapping population B493xQAL and 70349 using 212 polymorphic SNPs.       **Figure S1 – Number of contigs vs. length of contigs with hits to NCBI database.** Histogram of number of contigs with one or more hits to NCBI database using BLASTX vs. length of the contig sequence.     **Figure S2 – Genotype transcript contribution to the overall CAP3 assembly.**  Contribution of transcript sequences from each genotype (B493xQAL, B6274, B7262 and B493) to the overall CAP3 assembly.    **Figure S3 – Comparative analysis of carrot Sanger-based sequence genes and the corresponding EST contigs.** Comparative analysis of carrot Sanger-based sequence genes (A) and the corresponded EST contig (B) from our de novo assembly. The X-axis is the sequence base pair position and the Y-axis indicates read coverage. Different colors identify reads from three different genotypes as green: B493xQAL; yellow: B6274; and violet: B7262.      **Figure S4 - Intra- and inter-sample SNP distribution.** Intra and inter-sample polymorphism distribution of computationally detected SNPs among genotype at a depth of sequence coverage of 20. Inbred line order is B493xQAL, B6274 and B7262. * M=intra-sample monomorphic, inter-sample polymorphic;  P= intra and inter-sample polymorphic. Click here for file

Additional file 2 **Fasta file with 58,751 assembled sequences**. Click here for file

Additional file 3 **Table S3 - EST contigs related to transposable elements.** EST contigs related to transposable elements, as indicated by BLAST2GO annotation and their classification into subfamilies based on similarity to RepBase entries. Original annotation was maintained for contigs showing no significant similarity to RepBase.    **Table S5 - EST containing fragments of carrot Tdc transposons.** Characteristics of EST contigs containing fragments of carrot  Tdc transposons. Cells highlighted in yellow indicate elements showing  the highest similarity to the corresponding contigs.    **Table S6 - Characteristics of EST containing fragments of carrot DNA transposons DcMaster/Krak, DcSto, and Dc-hAT1.Table S10 - Information about SSR primers tested in this study.     Table S11 - Information about SNPs tested in this study.   ** Click here for file

Additional file 4 **Assembly methods and parameters**. Click here for file
