# Supplementary material for: De novo assembly and characterization of the carrot transcriptome reveals novel genes, new markers, and genetic diversity
Source: BMC Genomics. 2011 Aug 2;12:389. doi: 10.1186/1471-2164-12-389 (PMC3224100; doi:10.1186/1471-2164-12-389)
Supplement: Additional file 1 — Table S1 – Individual genotype transcriptome assemblies. Summary of the number of contigs and singletons obtained for the B493, B493×QAL, B6274 and B7262 individual transcriptome assemblies using different assembly methods. Table S2 – Combined transcriptome assemblies. Summary of the B493, B493×QAL, B6274, and B7262 combined transcriptome assemblies. Table S4 Transposable element superfamilies and families represented in ESTs.Table S7 - Distribution of motif length in the SSR dataset.Table S8 - Comparison of SNP validation rates using intron prediction.Table S9 - Polymorphic SNPs tested in two mapping populations. Summary of results obtained by screening of two mapping population B493xQAL and 70349 using 212 polymorphic SNPs. Figure S1 – Number of contigs vs. length of contigs with hits to NCBI database. Histogram of number of contigs with one or more hits to NCBI database using BLASTX vs. length of the contig sequence. Figure S2 – Genotype transcript contribution to the overall CAP3 assembly. Contribution of transcript sequences from each genotype (B493xQAL, B6274, B7262 and B493) to the overall CAP3 assembly. Figure S3 – Comparative analysis of carrot Sanger-based sequence genes and the corresponding EST contigs. Comparative analysis of carrot Sanger-based sequence genes (A) and the corresponded EST contig (B) from our de novo assembly. The X-axis is the sequence base pair position and the Y-axis indicates read coverage. Different colors identify reads from three different genotypes as green: B493xQAL; yellow: B6274; and violet: B7262. Figure S4 - Intra- and inter-sample SNP distribution. Intra and inter-sample polymorphism distribution of computationally detected SNPs among genotype at a depth of sequence coverage of 20. Inbred line order is B493xQAL, B6274 and B7262. * M=intra-sample monomorphic, inter-sample polymorphic; P= intra and inter-sample polymorphic. [file 1471-2164-12-389-S1.DOC]

**Supplementary Tables and Figures**

**Supplementary Tables**

Table S1 **Individual genotype transcriptome assemblies.** Page 2, this file

Table S2 **Combined transcriptome assemblies.** Page 2, this file

Table S3 **EST contigs related to transposable elements.** Additional File 3

Table S4 **Transposable element superfamilies and families represented in ESTs.** Page 2, this file

Table S5 **EST containing fragments of carrot Tdc transposons.** Additional File 3

Table S6 **Characteristics of EST containing fragments of carrot DNA transposons *DcMaster*/*Krak*, *DcSto*, and *Dc-hAT1*.** Additional File 3

Table S7 **Distribution of motif length in the SSR dataset.** Page 2, this file

Table S8 **Comparison of SNP validation rates using intron prediction.** Page 3, this file

Table S9 **Polymorphic SNPs tested in two mapping populations.** Page 3, this file

Table S10 **Information about SSR primers tested in this study.** Additional File 3

Table S11 **Information about SNPs tested in this study.** Additional File 3

**Supplementary Figures**

Figure S1 **Number of contigs vs. length of contigs with hits to NCBI database.**

Page 4, this file

Figure S2 **Genotype transcript contribution to the overall CAP3 assembly.** Page 4, this file

Figure S3 **Comparative analysis of carrot Sanger-based sequence genes and the corresponding EST contigs** Pages 5-15, this file

Figure S4 **Intra- and inter-sample SNP distribution.** Page 18, this file

Additional file 2: **Fasta file with 58,751 assembled sequences.**

Additional file 4:  **Assembly methods and parameters.**

**Supplementary Tables**

| **Table S1 Summary of the B493, B493×QAL, B6274 and B7262 individual genotype transcriptome assemblies** | | |
| --- | --- | --- |
| **Assembly method** | **Genotype** | **Number of contigs and singletons in output** |
| Sanger + CAP3 | B493 | 7,285 |
| Illumina+Velvet+CAP3 | B493xQAL | 39,901 |
| Illumina+Velvet+CAP3 | B6274 | 31,337 |
| Illumina+Velvet+CAP3 | B7262 | 34,218 |
| Illumina+ABySS | B493xQAL | 193,844 |
| Illumina+ABySS | B6274 | 133,933 |
| Illumina+ABySS | B7262 | 141,484 |
| Total |  | 582,002 |

| **Table S2 Summary of the carrots B493, B493×QAL, B6274, and B7262 combined transcriptome assemblies** | |
| --- | --- |
| Total number of contigs | 57,840 |
| Total number of singletons | 911 |
| Minimum length (nt) | 100 |
| Maximum length (nt) | 15,691 |
| Total Base pairs (nt) | 45,135,190 |
| Mean length (nt) | 768.2 |
| N50 (nt) | 1,378 |

| **Table S4 Transposable element superfamily and family represented in our ESTs** | | | | |
| --- | --- | --- | --- | --- |
| **Superfamily** | **TE family** | **EST number** | **e-value range** | |
| *PIF/Harbinger* | *DcMaster/Krak* | 9 | 8.00E-04 | 1.00E-108 |
| MITE*/Stowaway* | *DcSto* | 22 | 2.00E-03 | 1.00E-107 |
| *hAT* | *Dc-hAT1* | 15 | 7.00E-03 | 3.00E-20 |
| *En/Spm* | *Tdc* | 17 | 2.00E-05 | 1.00E-127 |

| **Table S7 Distribution of motif length in the carrot EST SSR dataset** | | |
| --- | --- | --- |
| **Motif length** | **Number of SSRs** | **Percentage** |
| 2 | 2,209 | 25 |
| 3 | 4,196 | 47.6 |
| 4 | 1,463 | 16.6 |
| 5 | 428 | 4.8 |
| 6 | 527 | 6 |

| **Table S8 Comparison of SNP validation rates using intron prediction** | | |
| --- | --- | --- |
| **Category** | **Without intron prediction** | **With intron prediction** |
| Number of primers designed | 354 | - |
| Number of candidate primers | - | 120 |
| Number of primers that amplified expected products | 162 | 91 |
| % of primers that amplifies expected products | 46 | 76 |

| **Table S9 Polymorphic SNPs tested in two mapping population. Summary of results obtained by screening of two mapping population B493xQAL and 70349 using 212 polymorphic SNPs.** | | | | |
| --- | --- | --- | --- | --- |
|  |  |  |  |  |
| **Mapping population** | **# of SNPs tested** | **# of SNPs polymorphic (%)** | **# of common polymorphic SNPs (%)** | **Total number of polymorphic SNPs (%)** |
| 493 x QAL | 212 | 48 (23) | 11 (5) | 87 (41) |
| 70349 | 212 | 50 (24) |

**Supplementary figures**


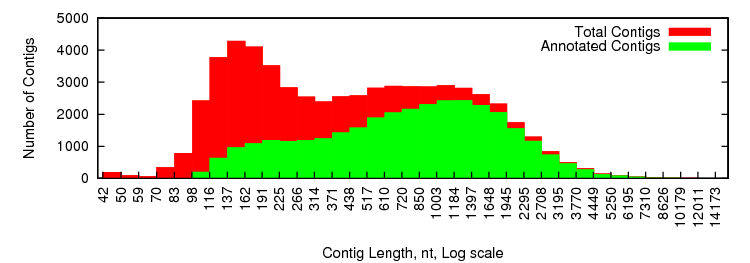
**Figure S1 Histogram of number of contigs with one or more hits to NCBI database using BLASTX vs length of the contig sequence**

**Figure S2 Contribution of transcript sequences from each carrot genotype (B493xQAL, B6274, B7262 and B493) in the overall CAP3 assembly**

**Figure S3 Comparative analysis of carrot Sanger-based sequence genes (A) and the corresponded EST contig (B) from our *de novo* assembly. The Y-axis indicates read coverage and the X-axis is the sequence nucleotide position. Different colors identify reads from three different genotypes as green: B493xQAL; yellow: B6274; and violet: B7262**


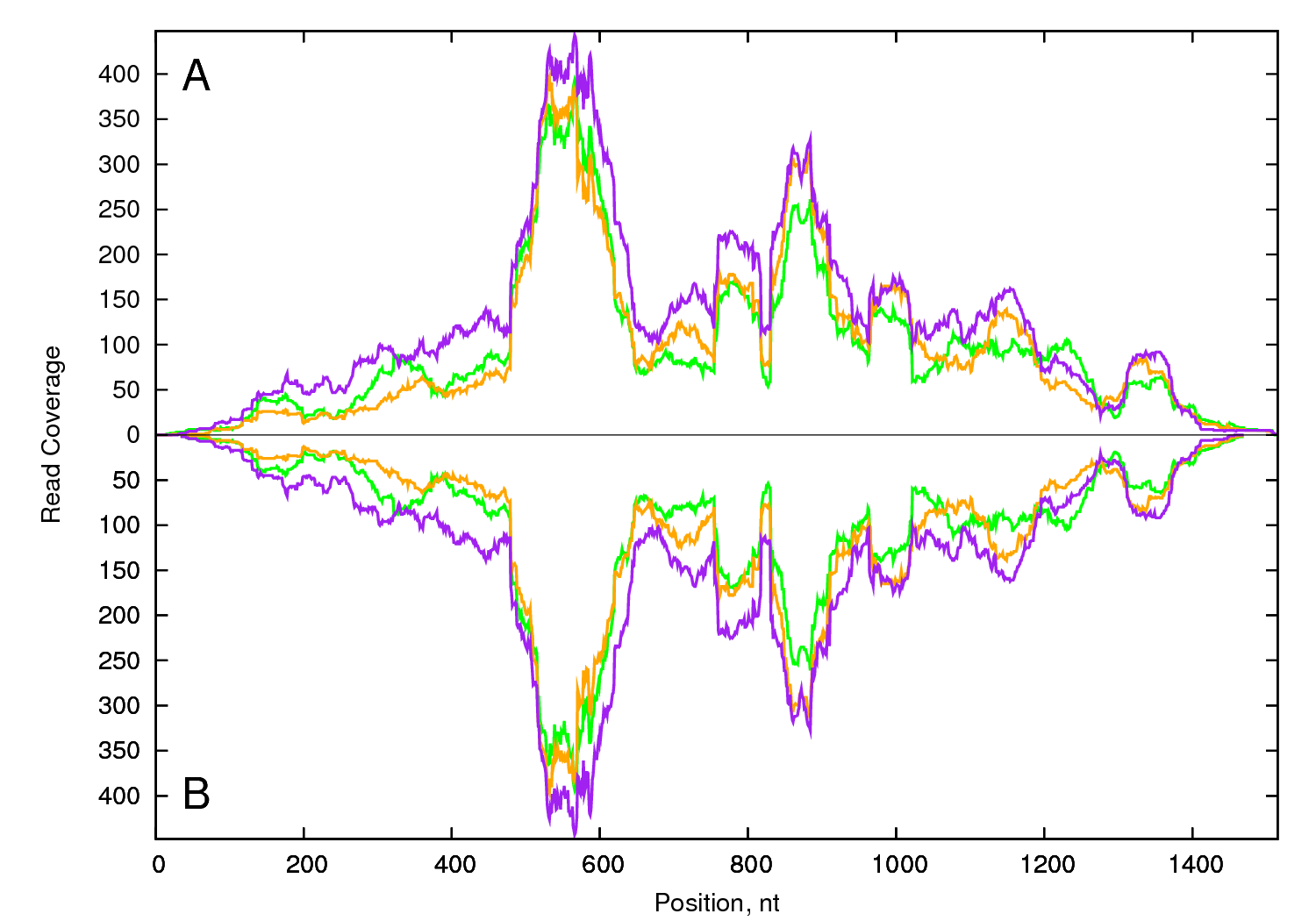


**A: PSY01 GenBank DQ192186 B: EST contig654**

**A: PSY02 GenBank DQ192187 B: EST contig8137**


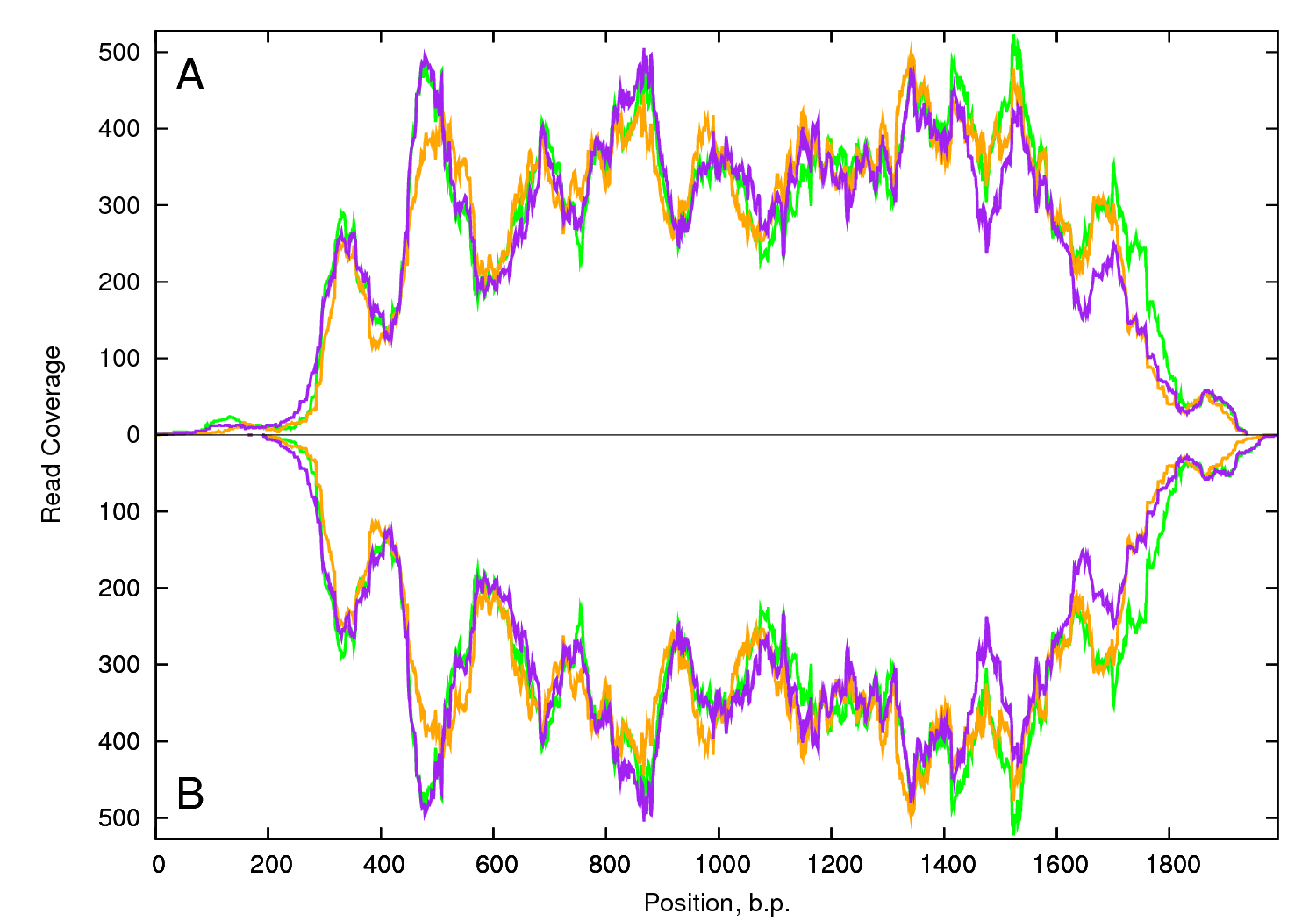


**A**: **LRR-S-T01 GenBank AB178084.2 B: EST contig27188**


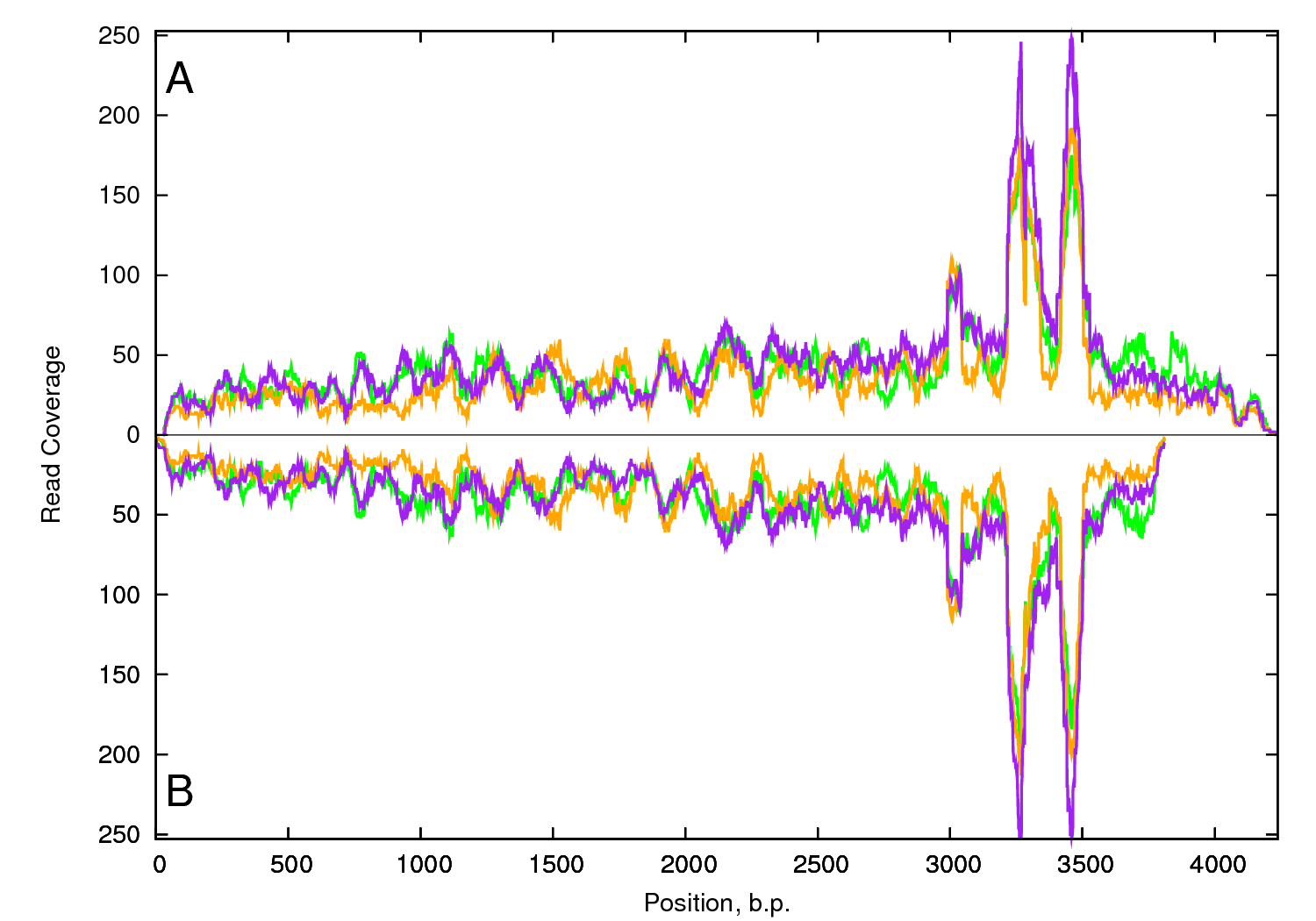


**A: PDS01 GenBank DQ222429 B: EST contig792**


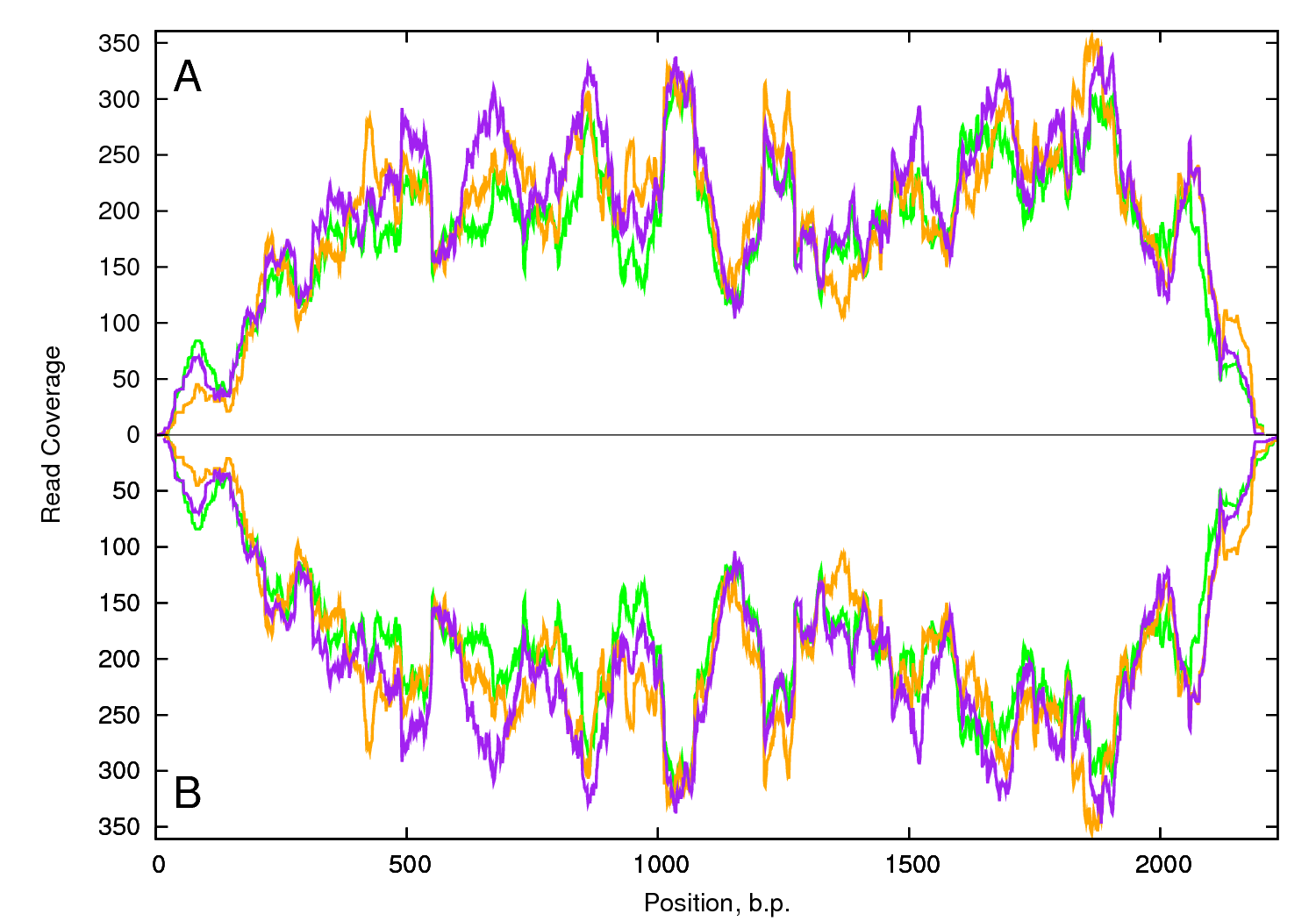


**A: LCYE01 GenBank DQ192192 B: EST contig36415**


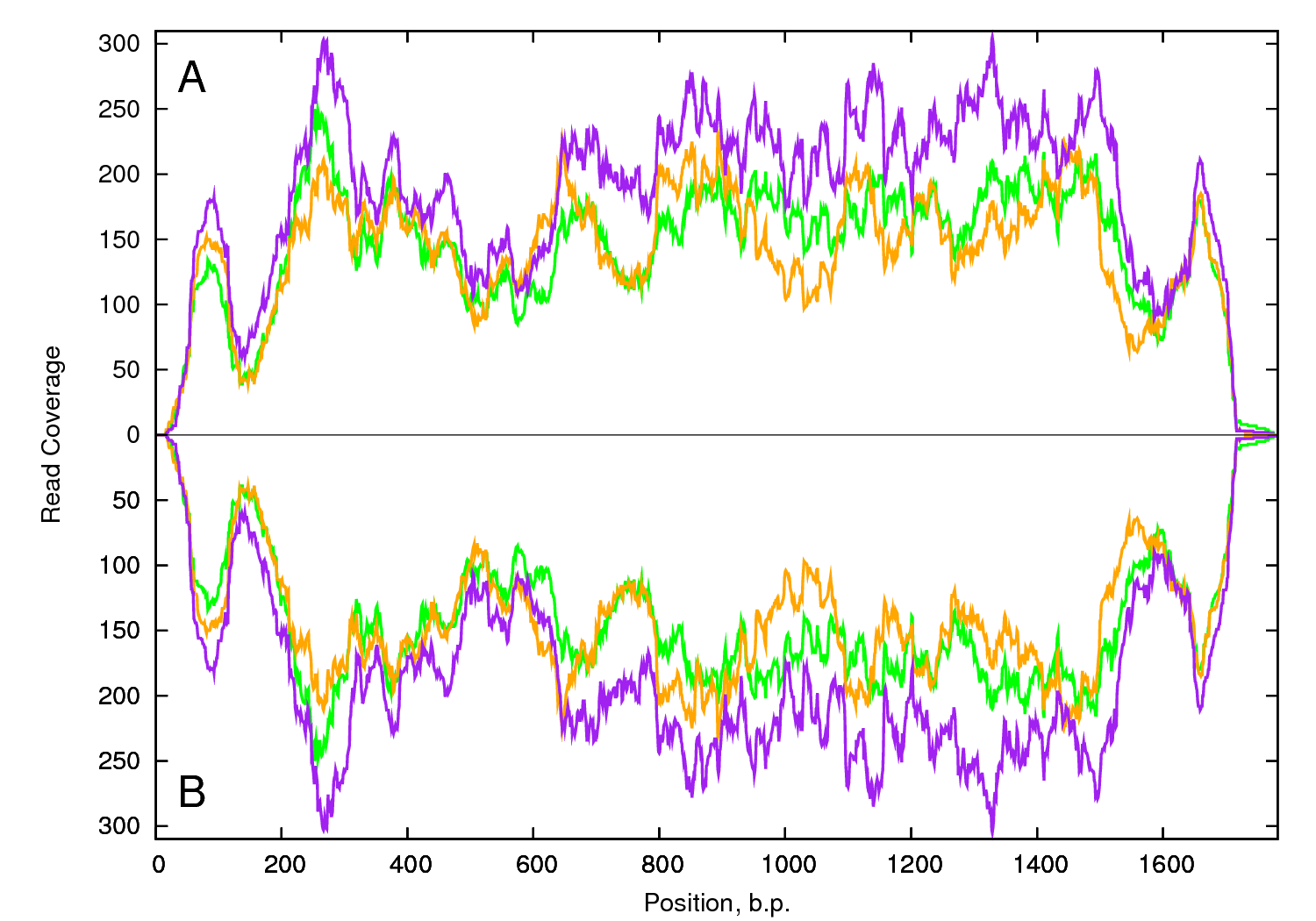


**A: LCYB01 GenBank DQ192190 B: EST contig15926**


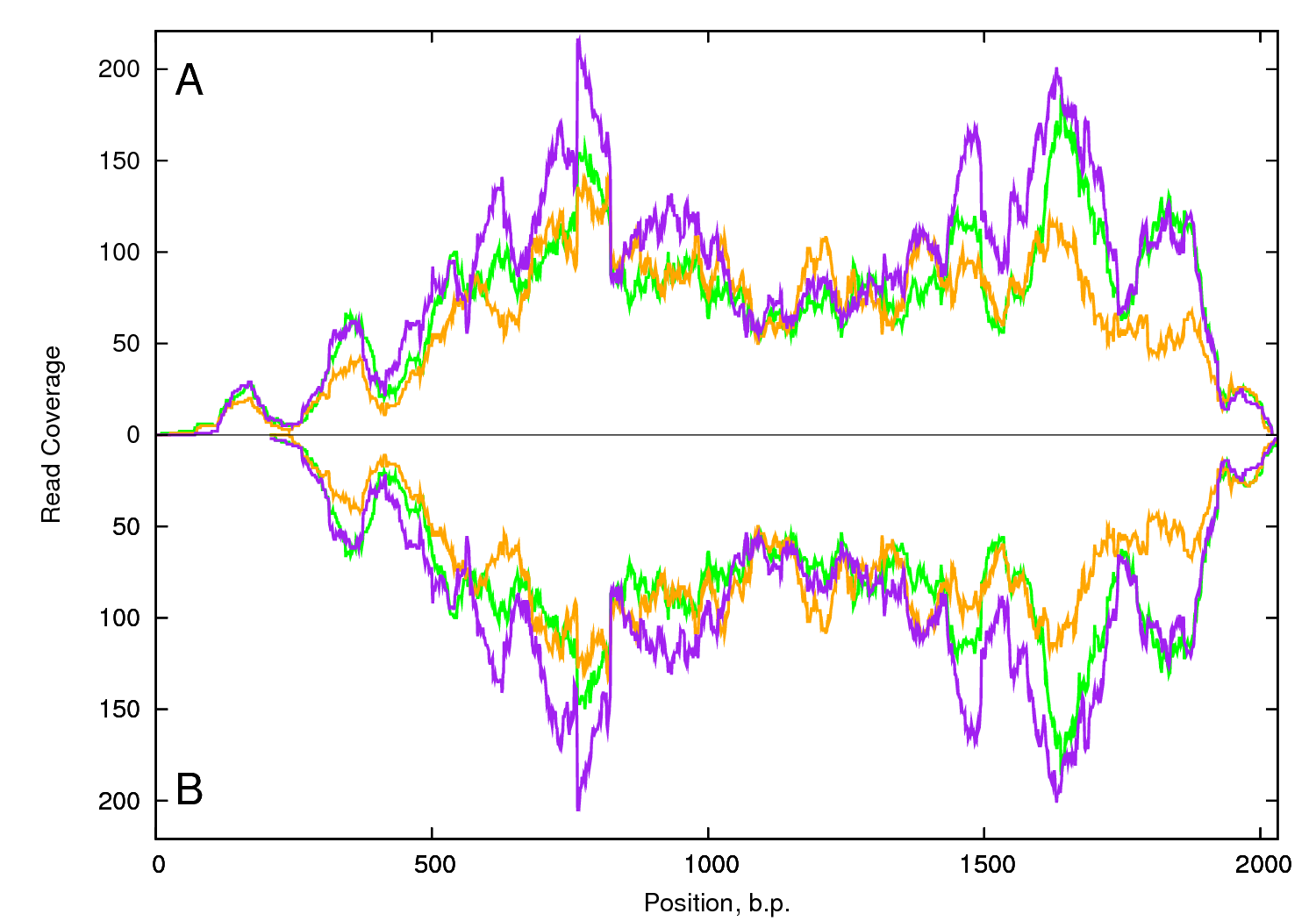


**A: DAREP2LPT01 GenBank M64746 B: EST contig9644**


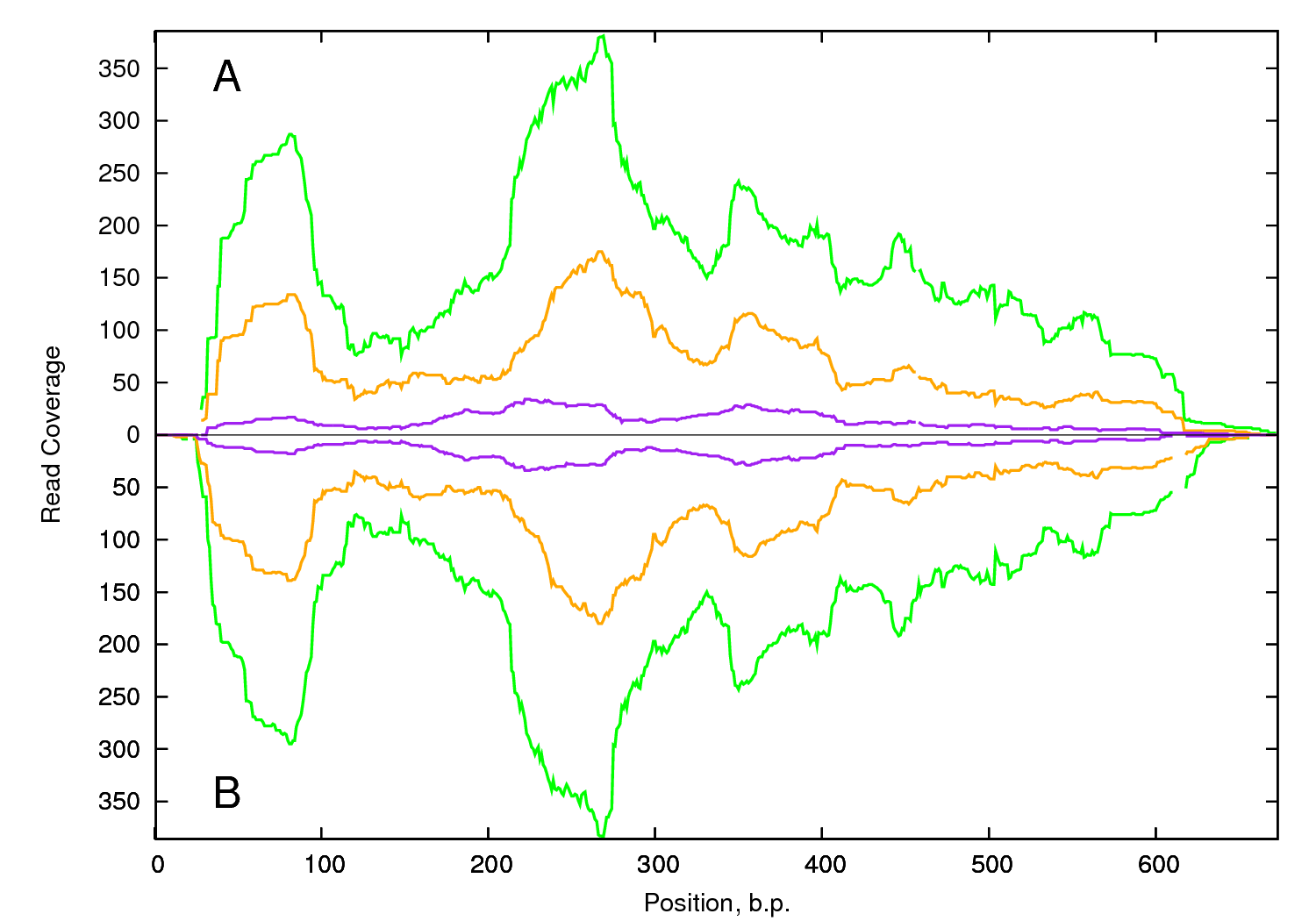


**A: DcADH01 GenBank AB104856.2 B: EST contig49498**


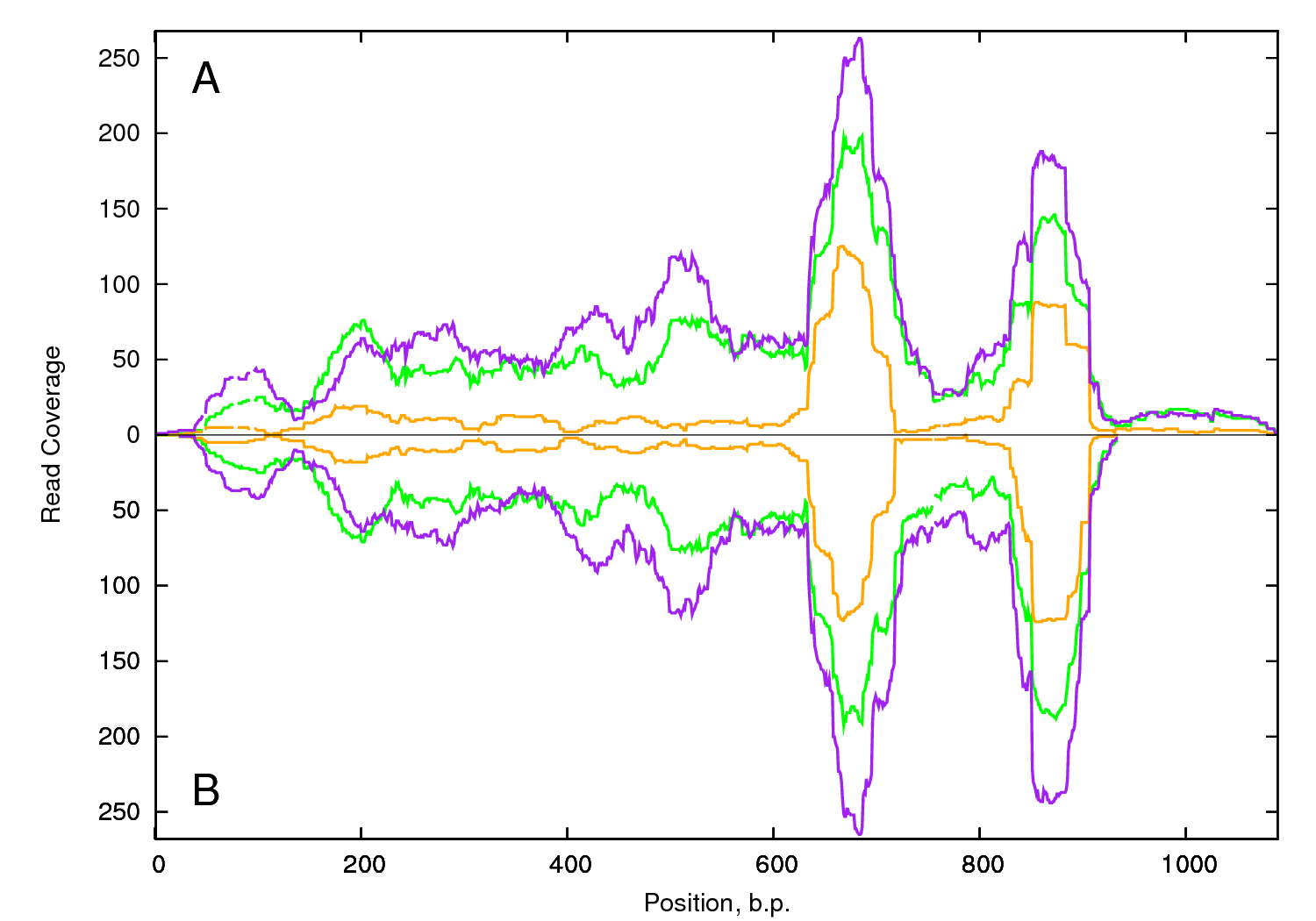


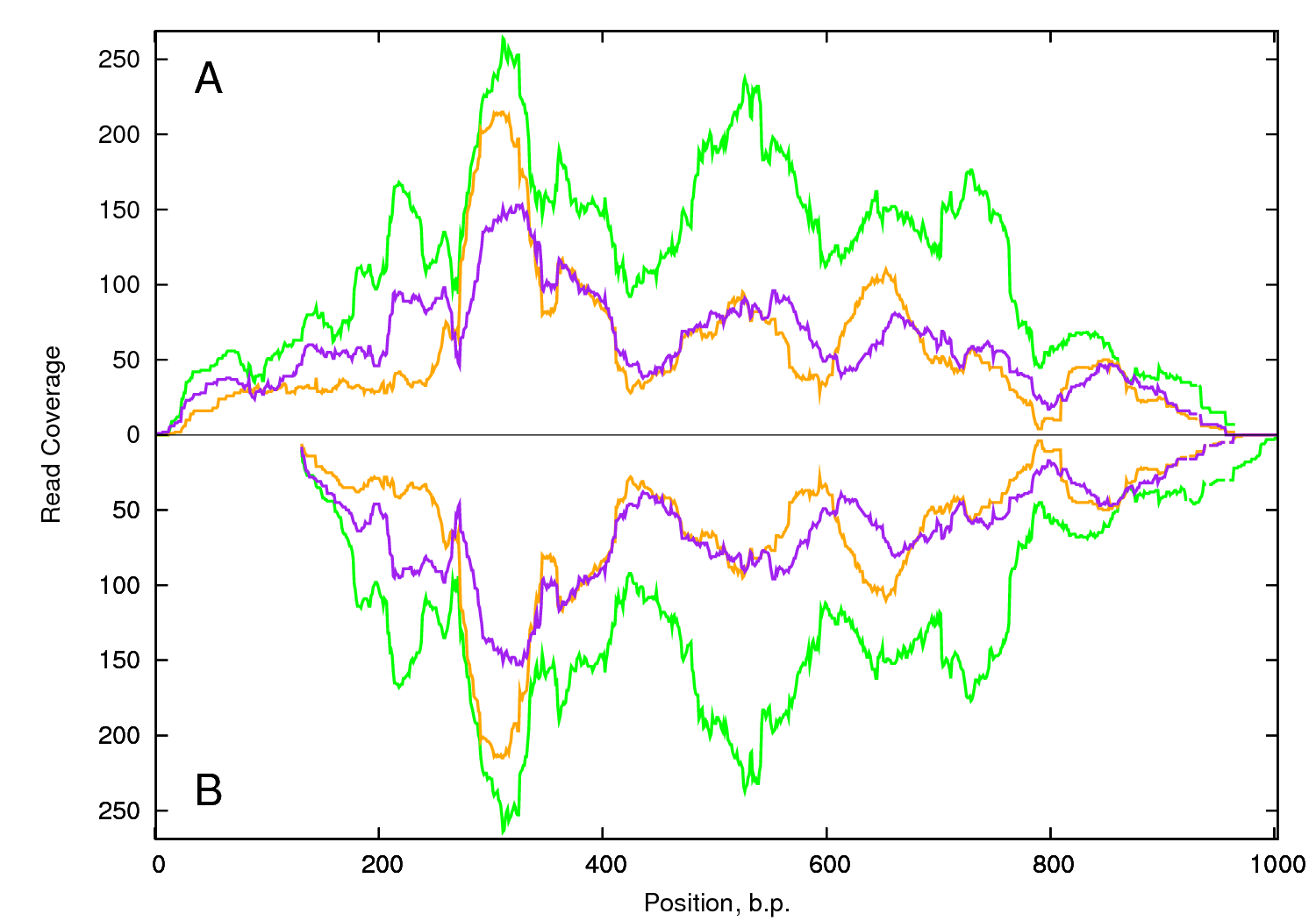


**A: Dcarg-101 GenBank AB017501.1 B: EST contig23985**

**A: DcERF201 GenBank DQ192187 B: EST contig595**


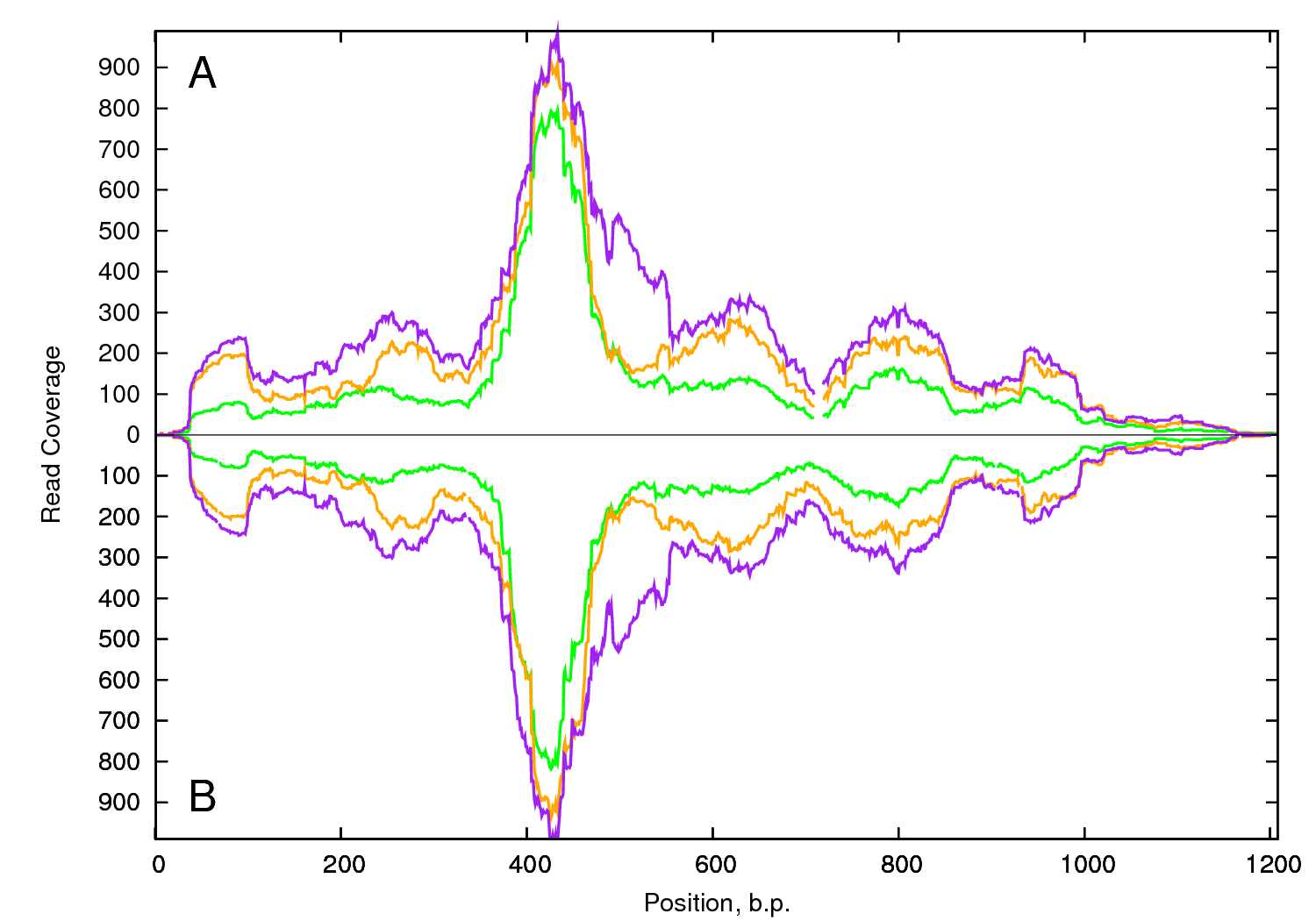


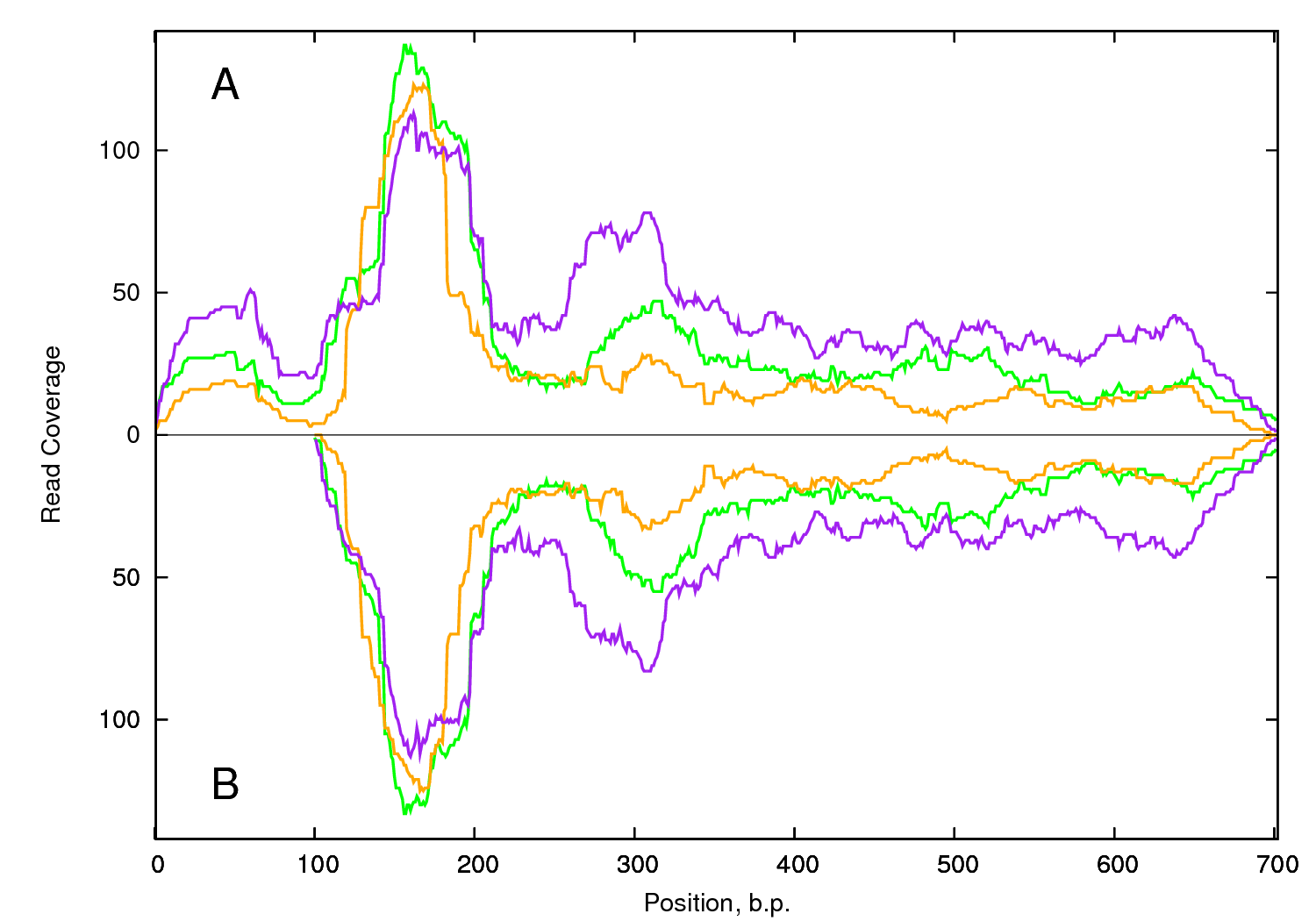


**A: DcMYB101 GenBank AB218778.1 B: EST contig17550**

**A: DcPRP01 GenBank AB127961.1 B: EST contig46959**


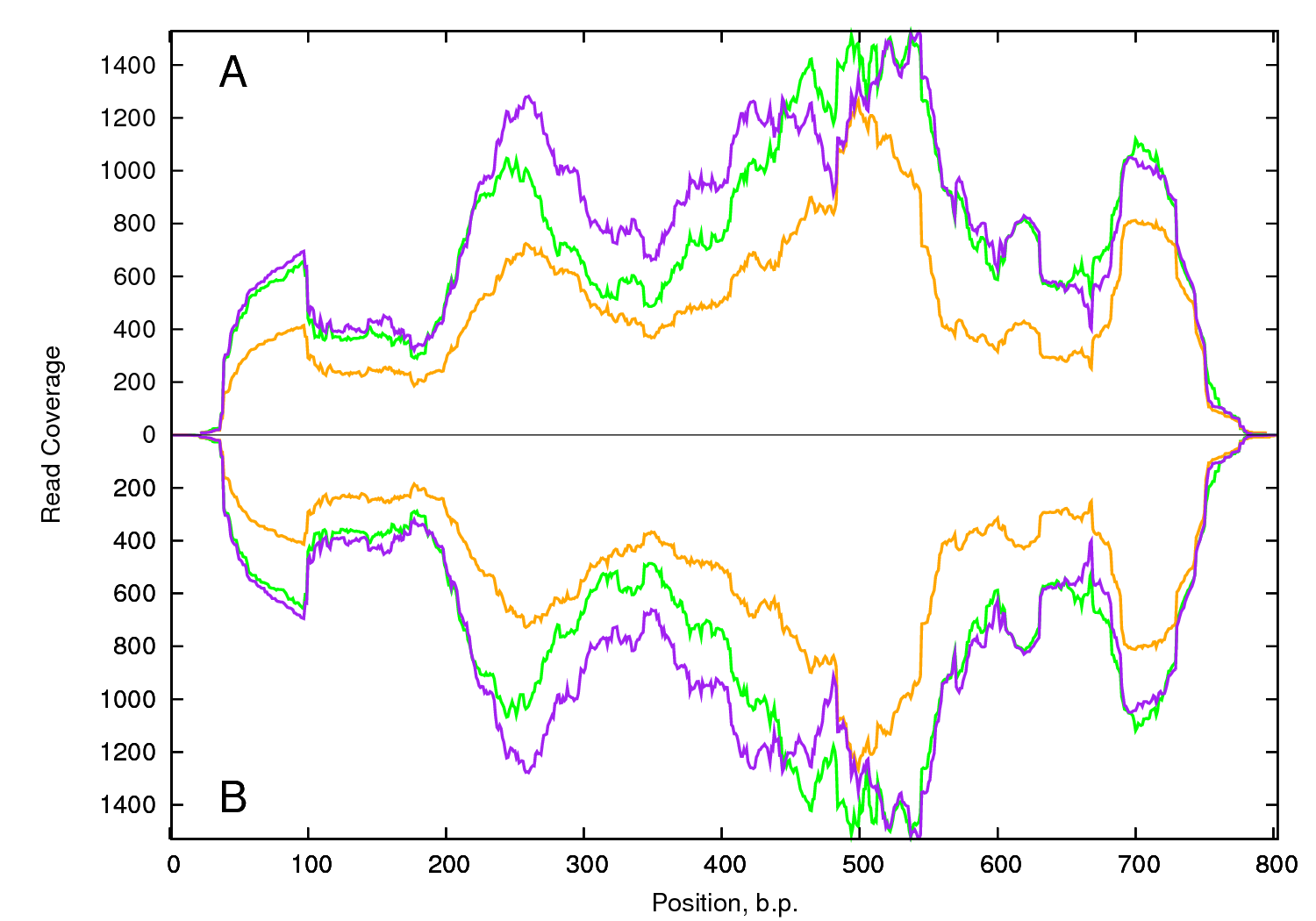


**A: FEN101 GenBank AB326233.1 B: EST contig9422**


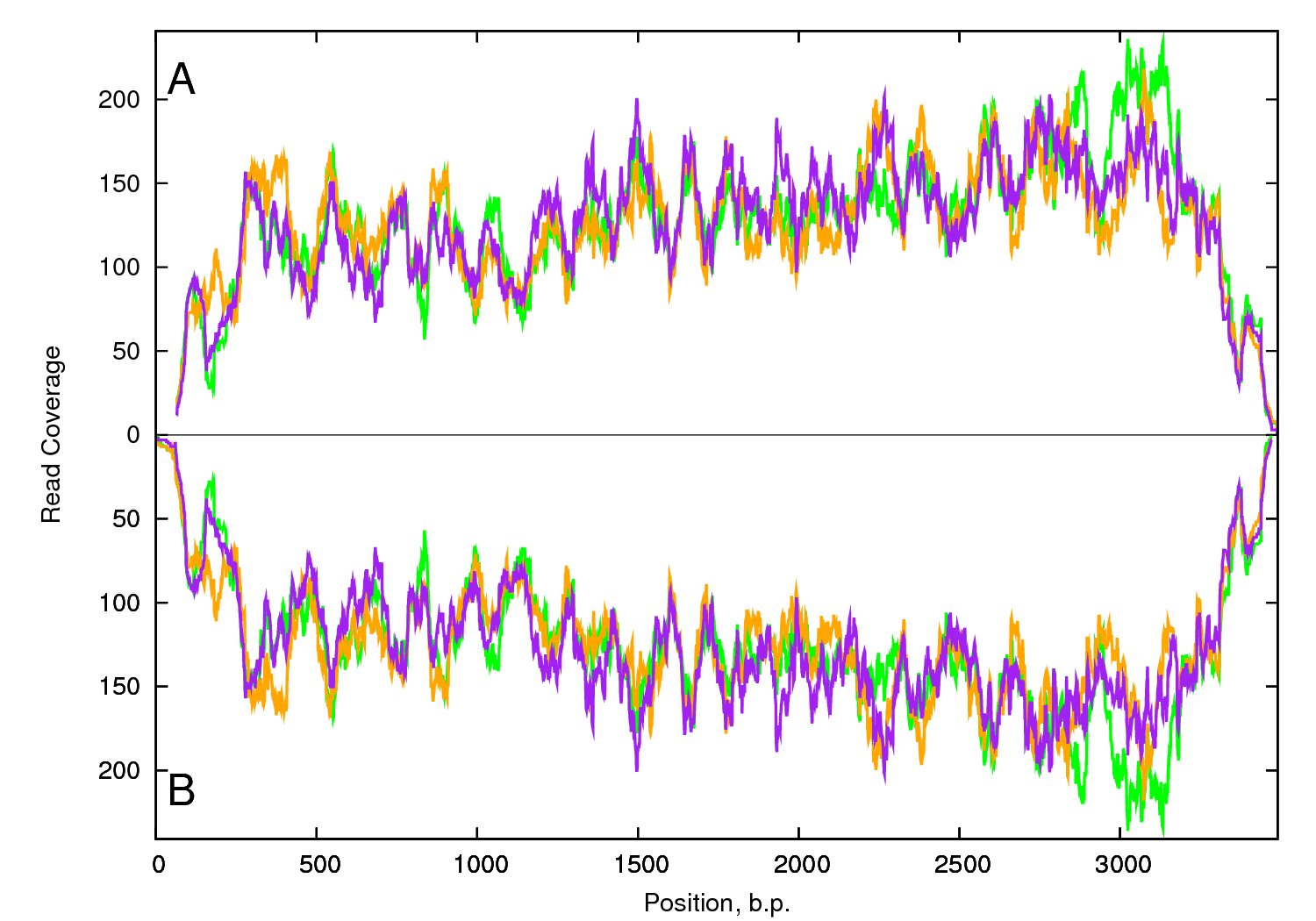


**A: TBA01 GenBank AY007250 B: EST contig14017**


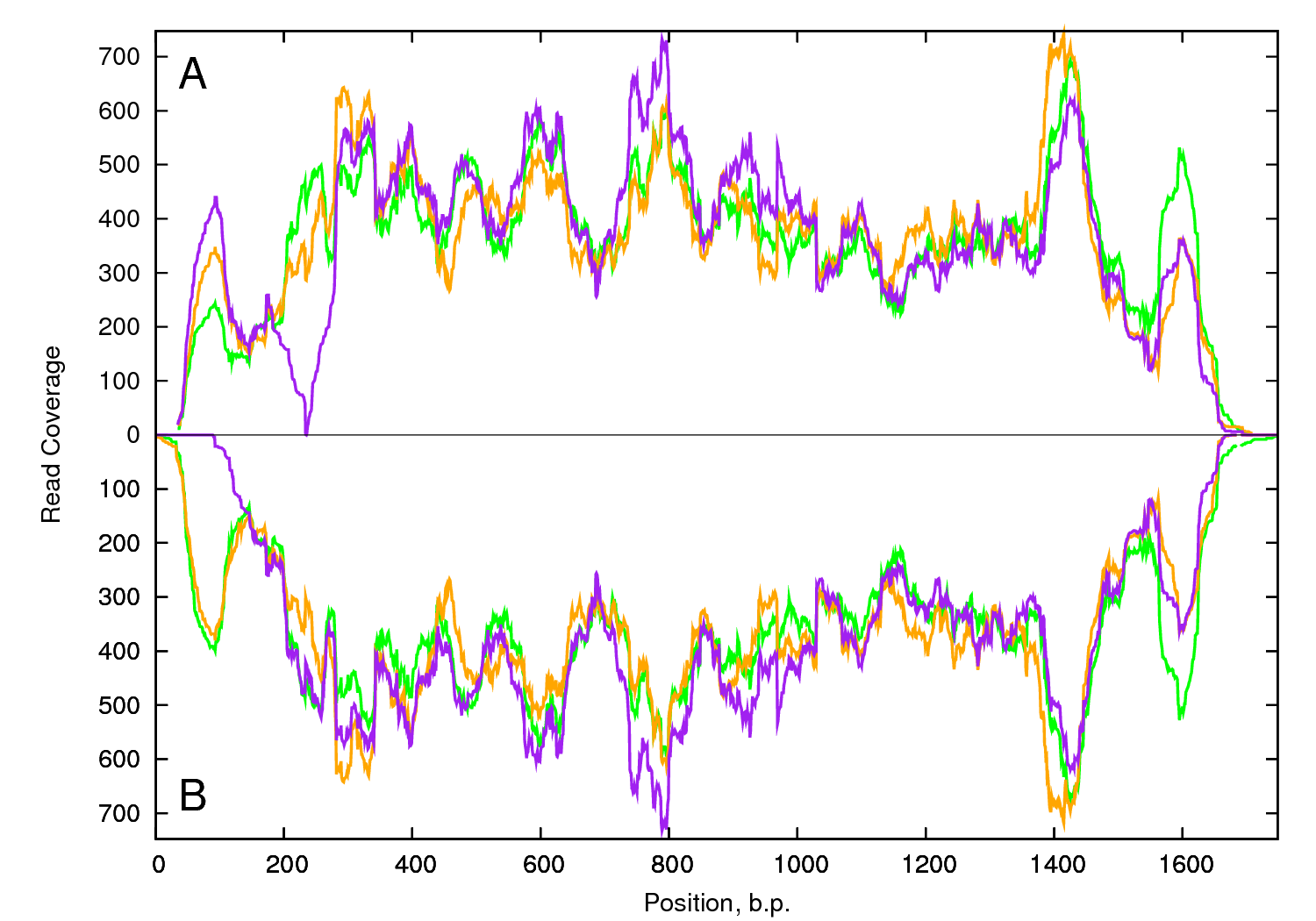


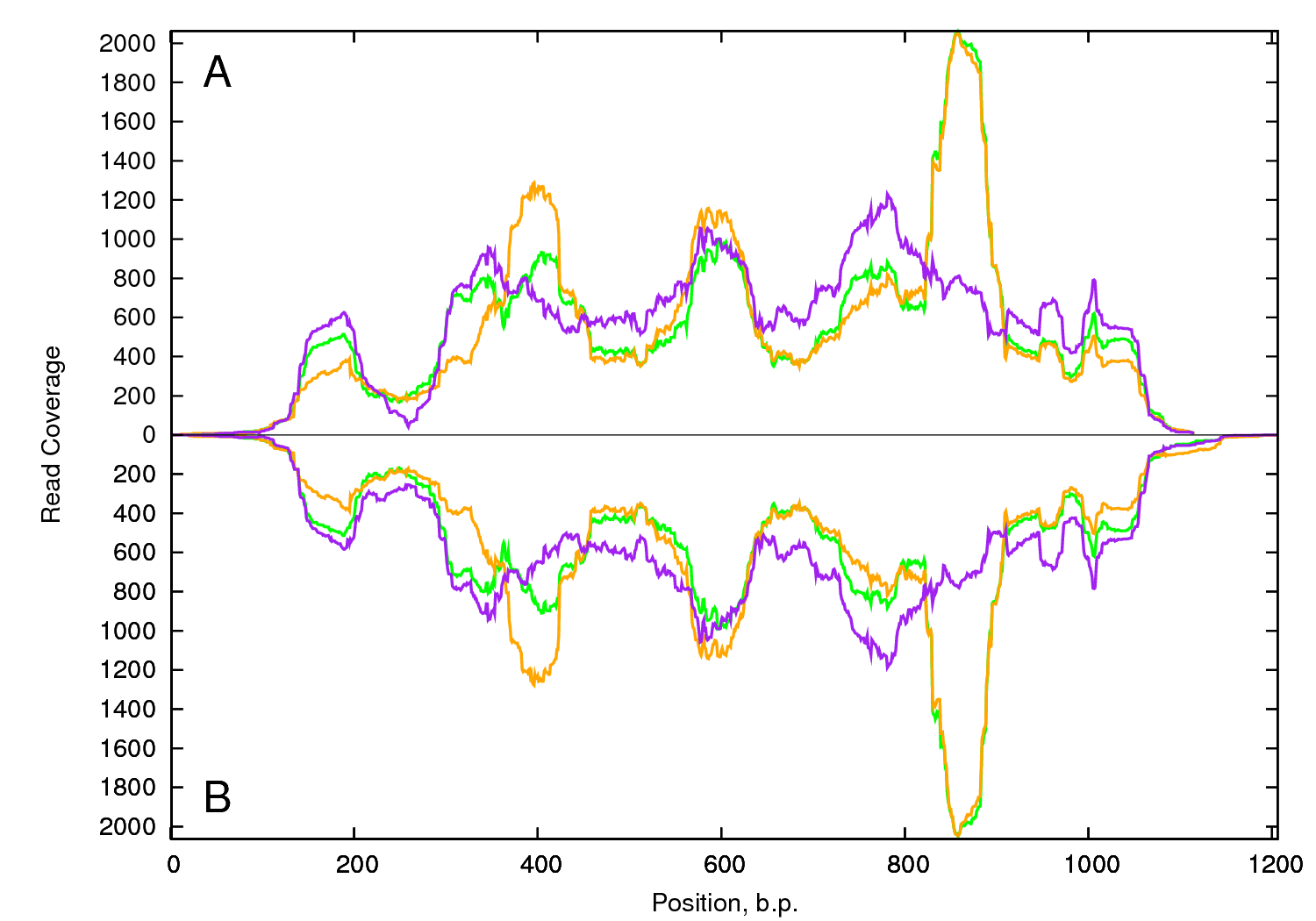


**A: cab301 GenBank AF207690.1 B: EST contig17323**

**A: CAPSE01 GenBank AB1262255.1 B: EST contig14777**


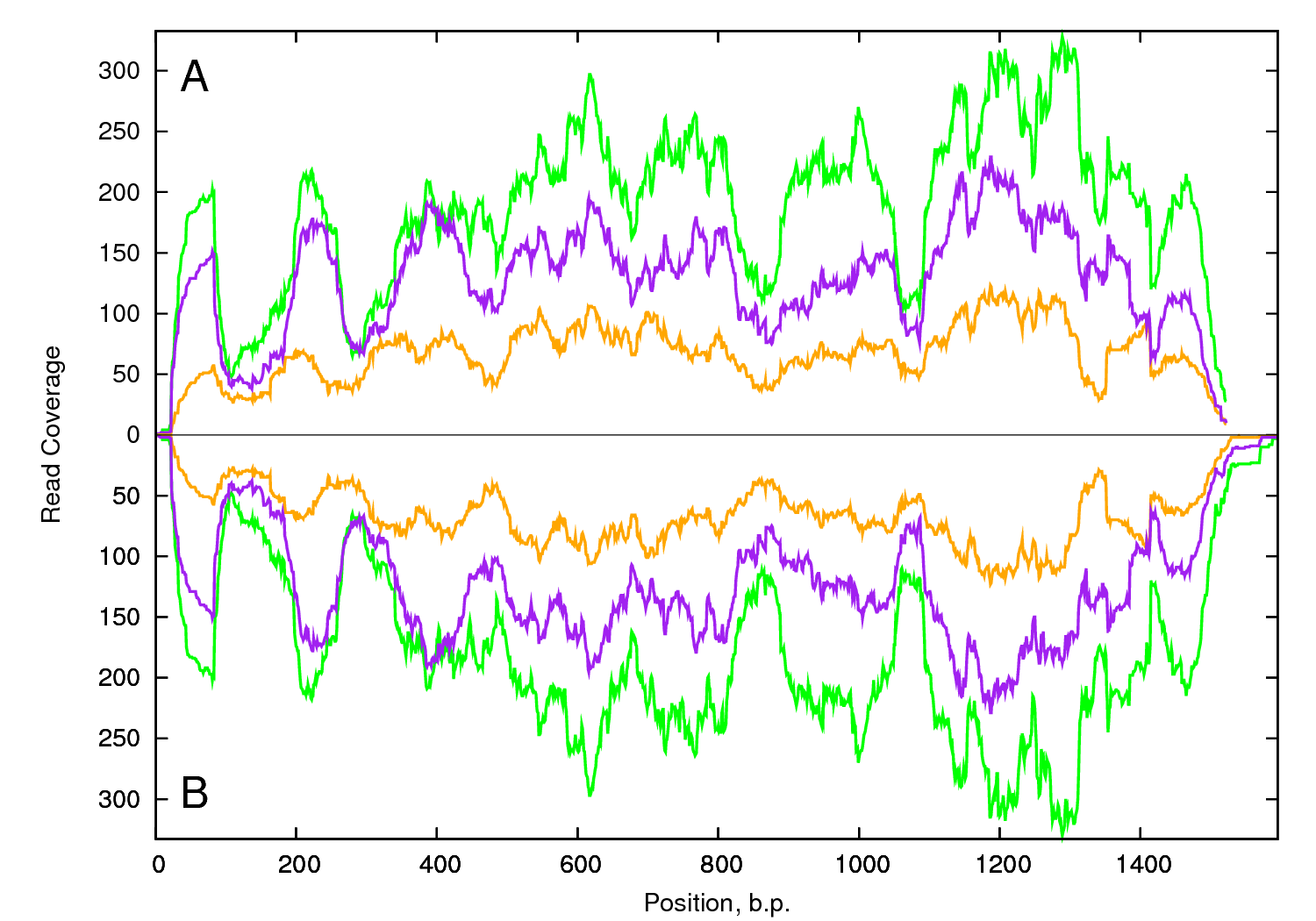


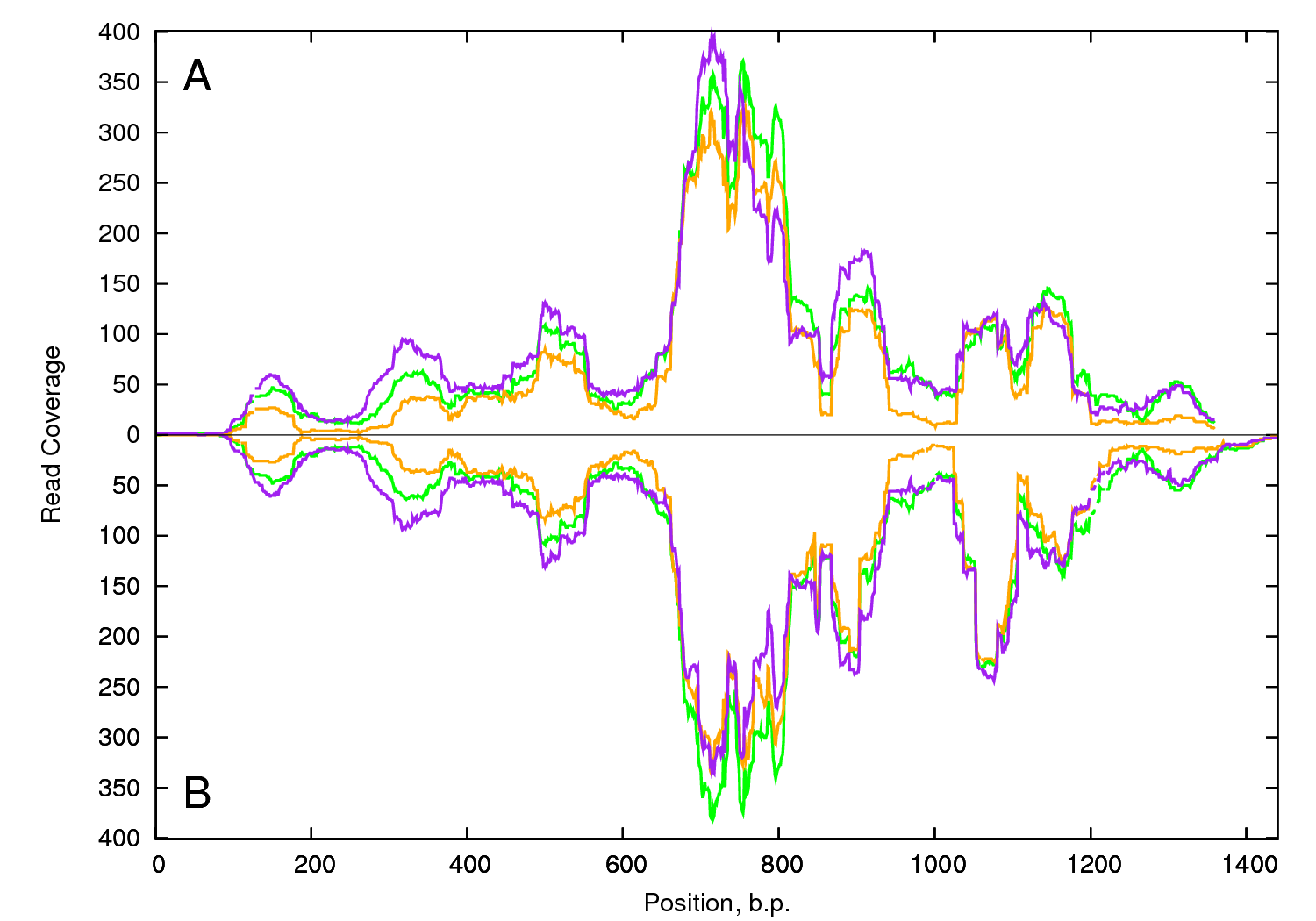


**A: DARAX11001 GenBank D14605.1 B: EST contig55312**

**A: NMCP101 GenBank D64087.1 B: EST contig5281**


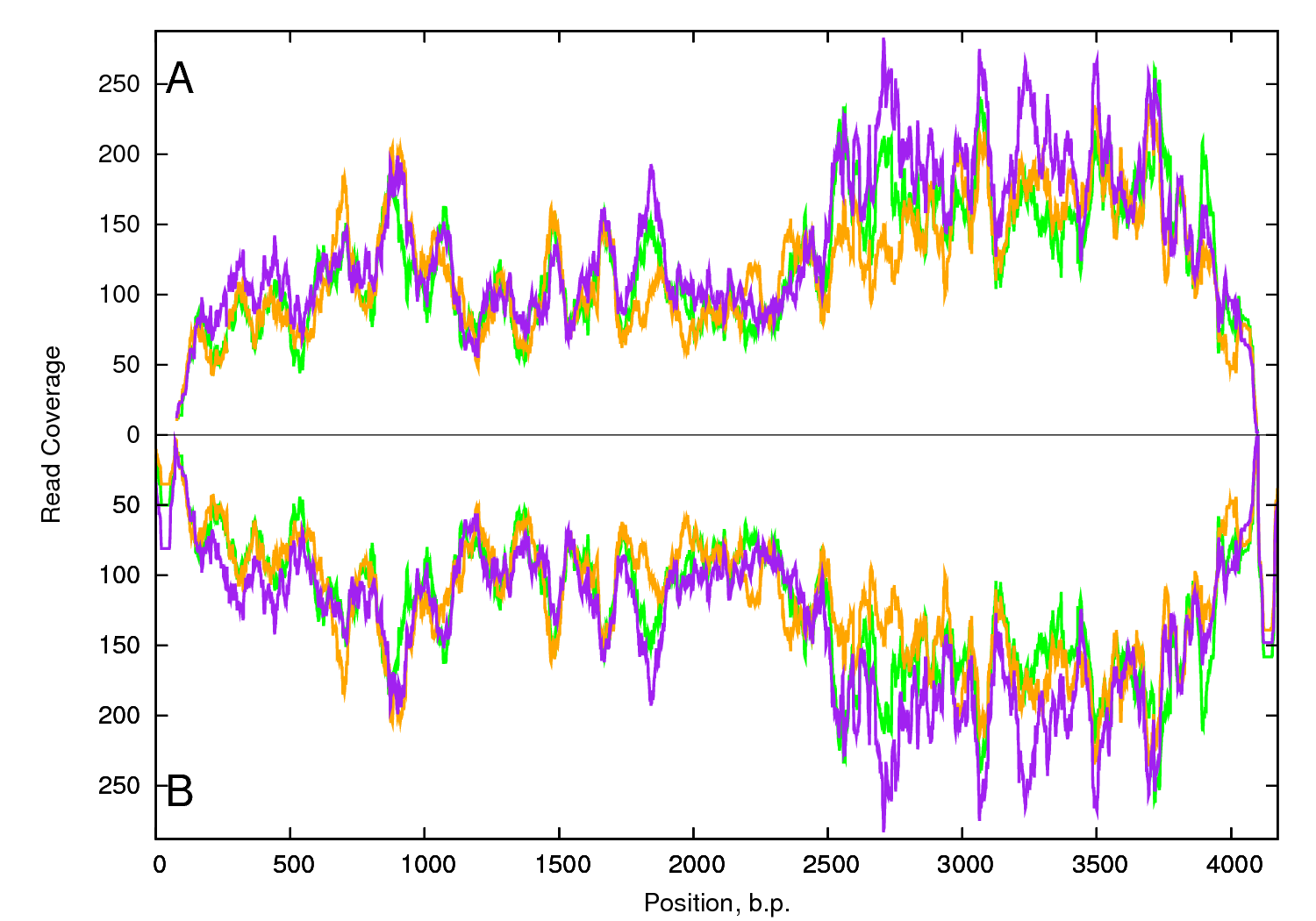


**A: P4001 GenBank AB012702.1 B: EST contig31355**


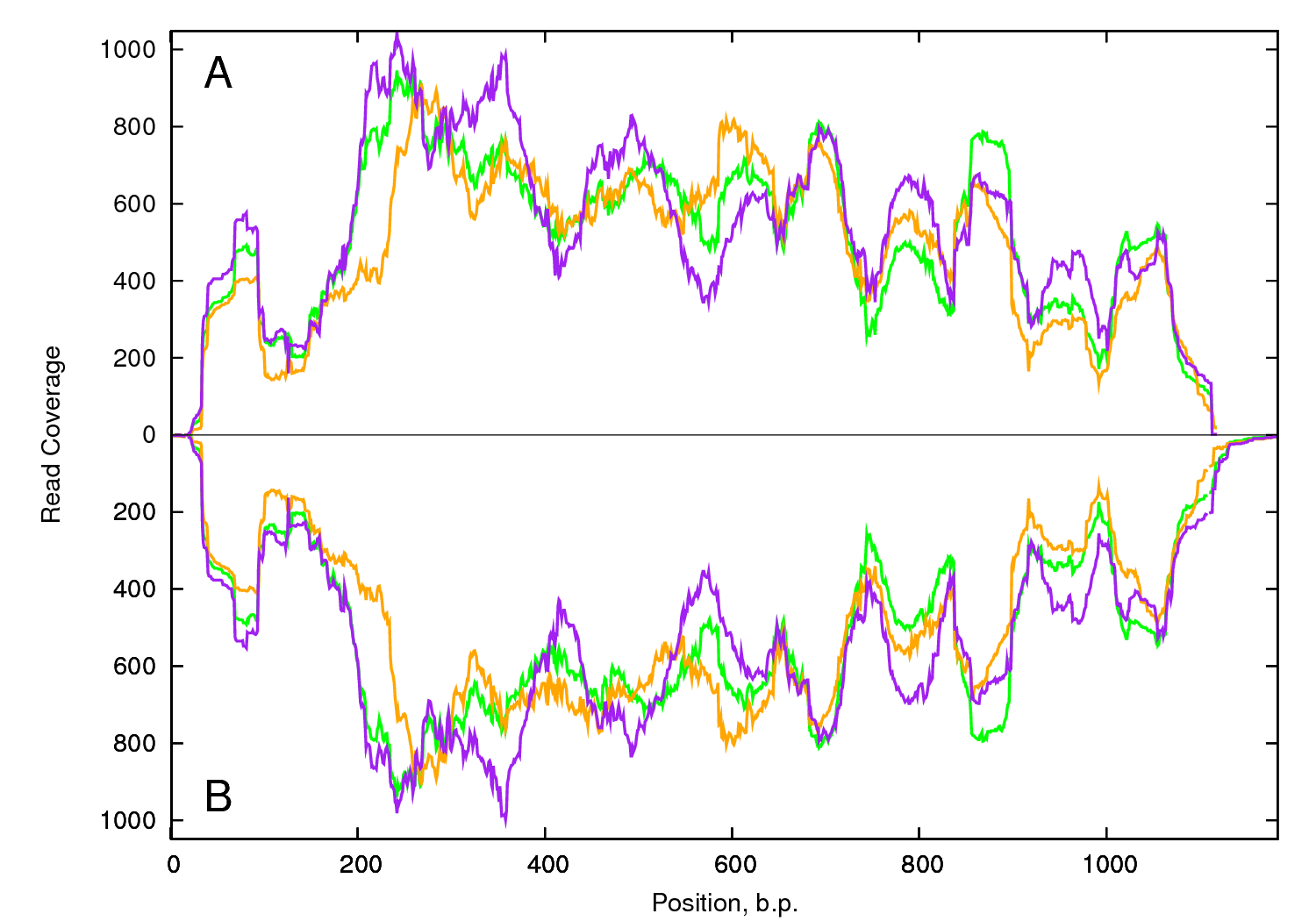


**A: CCS01 GenBank DQ192191.1 B: EST contig14661**


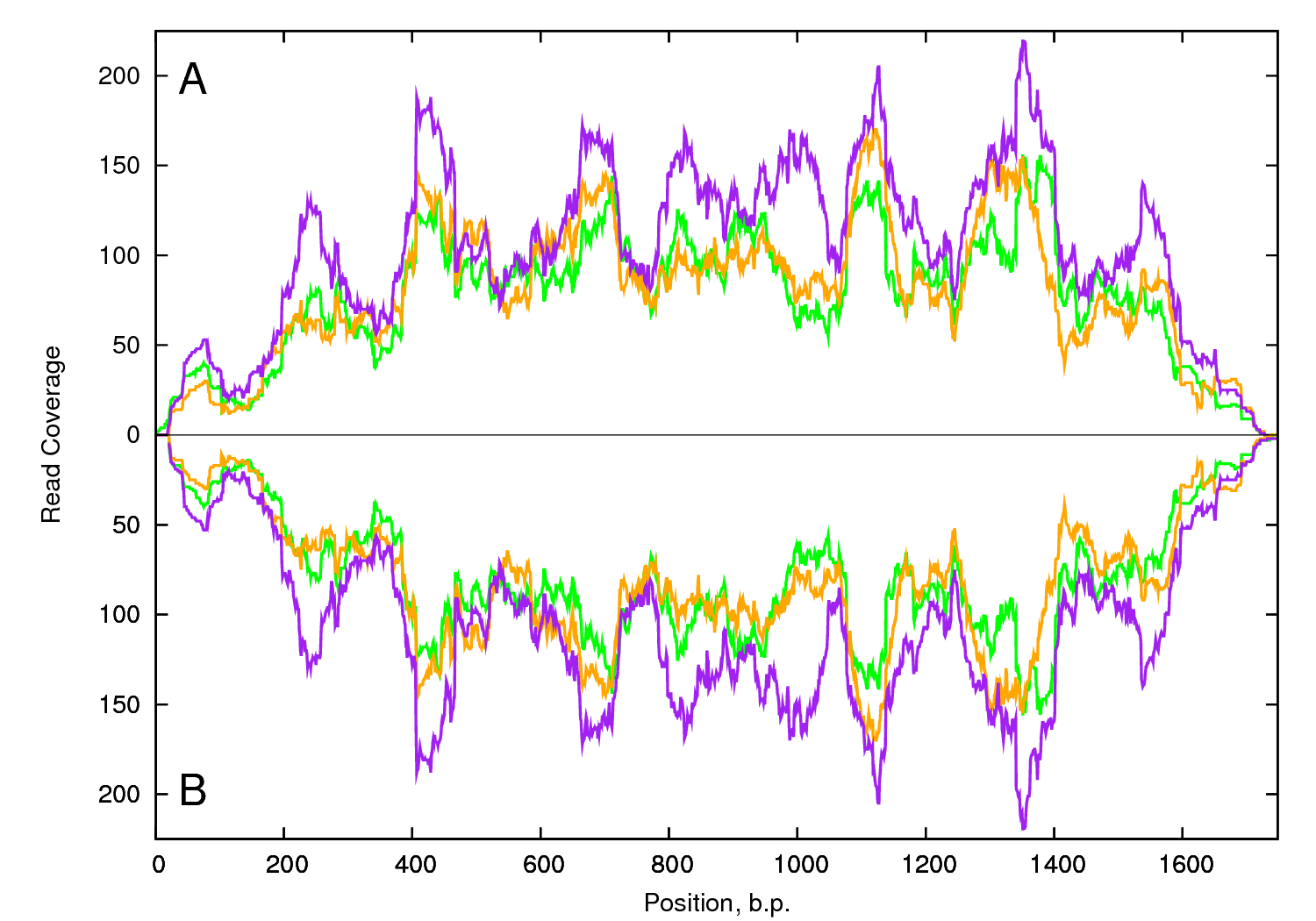


**Figure S4 Intra and inter-sample polymorphism distribution of computationally detected SNPs among genotype at a depth of sequence coverage of 20. Inbred line order is B493xQAL, B6274 and B7262**

*** M=intra-sample monomorphic, inter-sample polymorphic; P= intra and inter-sample polymorphic**
