# Supplementary material for: De novo assembly and characterization of the carrot transcriptome reveals novel genes, new markers, and genetic diversity
Source: BMC Genomics. 2011 Aug 2;12:389. doi: 10.1186/1471-2164-12-389 (PMC3224100; doi:10.1186/1471-2164-12-389)
Supplement: Additional file 4 — Assembly methods and parameters. [file 1471-2164-12-389-S4.DOC]

**Additional file 4** Assembly methods and parameters.

- Sanger Assembly
  1. Sanger read basecalls and quality scores were made with **phred** version 0.020425.c
     [http://www.phrap.org/phredphrapconsed.html#block_phred](http://www.phrap.org/phredphrapconsed.html#block_phred)
  2. Vector sequence ([pDNR-LIB](http://cuke.vcru.wisc.edu/simonlab/est/pDNR-LIB.txt)) and low quality bases were trimmed with **lucy** version 1.19p
     <ftp://ftp.tigr.org/pub/software/Lucy/>
  3. Resulting sequences and quality files were assembled with **CAP3** version 12/21/07 with default parameters
     <http://deepc2.psi.iastate.edu/aat/cap/capdoc.html>
  4. Resulting contigs and singlets were passed into the final reference assembly step below
- Illumina Assembly with Velvet
  The following steps were performed separately for each of the three genotypes: B493×QAL, B6274, and B7262
  1. Three versions of the original Illumina reads were created to be used for assembly:
     - A. Unmodified original reads
     - B. 10 base pairs were trimmed from the left (5') end of every sequence
     - C. 10 base pairs were trimmed from the right (3') end of every sequence
  2. Optimal kmer length was determined by performing assemblies on one genotype, B493×QAL, for varying odd kmer lengths from 23 to 59 b.p.
  3. Each of the three Illumina read sets was assembled separately using **Velvet** version 0.7.55
     <http://www.ebi.ac.uk/~zerbino/velvet/>
     using the determined optimal kmer length of 41 b.p.
     minimum length parameter of 50 b.p.
     insert length parameter of 232 b.p.
  4. The resulting three assemblies were merged by assembling with **CAP3** version 12/21/07 with default parameters
     <http://deepc2.psi.iastate.edu/aat/cap/capdoc.html>
  5. Resulting contigs were passed into the final reference assembly step below
- Illumina Assembly with ABySS
  The following steps were performed separately for each of the three genotypes: B493×QAL, B6274, and B7262
  1. The untrimmed Illumina reads (Set "A" from Velvet assembly above) were used
  2. Optimal kmer length was determined by performing assemblies on one genotype, B493×QAL, for varying odd and even kmer lengths from 22 to 60 b.p.
  3. Each of the three Illumina read sets was assembled separately using **ABySS** version 1.0.15 <http://www.bcgsc.ca/platform/bioinfo/software/abyss>
     using the determined optimal kmer length of 43 b.p.
     -e or --erode parameter = 2 (*i.e.* erode bases at the ends of blunt contigs with coverage less than this threshold)
     -E or --erode-strand parameter = 0
     -c or --coverage parameter = 2 (*i.e.* minimum coverage threshold)
  4. Resulting contigs were passed into the final reference assembly step below
- Reference Assembly ([Assembly 1](http://cuke.vcru.wisc.edu/simonlab/est/solexa/assembly1))
  1. **CAP3** version 12/21/07, with default parameters,
     <http://deepc2.psi.iastate.edu/aat/cap/capdoc.html>
     was used to assemble the 7 prior assemblies:
     - Sanger CAP3 contigs + singlets
     - Velvet assembly of B493×QAL
     - ABySS assembly of B493×QAL
     - Velvet assembly of B6274
     - ABySS assembly of B6274
     - Velvet assembly of B7262
     - ABySS assembly of B7262
  2. This assembly containing 59,493 sequences was named [Assembly 1](http://cuke.vcru.wisc.edu/simonlab/est/solexa/assembly1)
